# Leveraging chromatin state transitions for the identification of regulatory networks orchestrating heart regeneration

**DOI:** 10.1093/nar/gkae085

**Published:** 2024-02-14

**Authors:** Julio Cordero, Adel Elsherbiny, Yinuo Wang, Lonny Jürgensen, Florian Constanty, Stefan Günther, Melanie Boerries, Joerg Heineke, Arica Beisaw, Florian Leuschner, David Hassel, Gergana Dobreva

**Affiliations:** Department of Cardiovascular Genomics and Epigenomics, European Center for Angioscience (ECAS), Medical Faculty Mannheim, Heidelberg University, Mannheim, Germany; German Centre for Cardiovascular Research (DZHK), Partner Site Heidelberg/Mannheim, 69120 Heidelberg, Germany; Department of Cardiovascular Genomics and Epigenomics, European Center for Angioscience (ECAS), Medical Faculty Mannheim, Heidelberg University, Mannheim, Germany; Department of Cardiovascular Genomics and Epigenomics, European Center for Angioscience (ECAS), Medical Faculty Mannheim, Heidelberg University, Mannheim, Germany; Department of Cardiology, Angiology and Pneumology, University Hospital Heidelberg, 69120 Heidelberg, Germany; Institute of Experimental Cardiology, University Hospital Heidelberg, 69120 Heidelberg, Germany; German Centre for Cardiovascular Research (DZHK), Partner Site Heidelberg/Mannheim, 69120 Heidelberg, Germany; Max-Planck-Institute for Heart and Lung Research, Bad Nauheim, Germany; Institute of Medical Bioinformatics and Systems Medicine, Medical Center-University of Freiburg, Faculty of Medicine, University of Freiburg, 79110 Freiburg, Germany; German Cancer Consortium (DKTK), Partner site Freiburg, a partnership between DKFZ and Medical Center - University of Freiburg, 69110 Heidelberg, Germany; Department of Cardiovascular Physiology, European Center for Angioscience (ECAS), Medical Faculty Mannheim, Heidelberg University, Mannheim, Germany; German Centre for Cardiovascular Research (DZHK), Partner Site Heidelberg/Mannheim, 69120 Heidelberg, Germany; Institute of Experimental Cardiology, University Hospital Heidelberg, 69120 Heidelberg, Germany; German Centre for Cardiovascular Research (DZHK), Partner Site Heidelberg/Mannheim, 69120 Heidelberg, Germany; Department of Cardiology, Angiology and Pneumology, University Hospital Heidelberg, 69120 Heidelberg, Germany; German Centre for Cardiovascular Research (DZHK), Partner Site Heidelberg/Mannheim, 69120 Heidelberg, Germany; Department of Cardiology, Angiology and Pneumology, University Hospital Heidelberg, 69120 Heidelberg, Germany; German Centre for Cardiovascular Research (DZHK), Partner Site Heidelberg/Mannheim, 69120 Heidelberg, Germany; Department of Cardiovascular Genomics and Epigenomics, European Center for Angioscience (ECAS), Medical Faculty Mannheim, Heidelberg University, Mannheim, Germany; German Centre for Cardiovascular Research (DZHK), Partner Site Heidelberg/Mannheim, 69120 Heidelberg, Germany

## Abstract

The limited regenerative capacity of the human heart contributes to high morbidity and mortality worldwide. In contrast, zebrafish exhibit robust regenerative capacity, providing a powerful model for studying how to overcome intrinsic epigenetic barriers maintaining cardiac homeostasis and initiate regeneration. Here, we present a comprehensive analysis of the histone modifications H3K4me1, H3K4me3, H3K27me3 and H3K27ac during various stages of zebrafish heart regeneration. We found a vast gain of repressive chromatin marks one day after myocardial injury, followed by the acquisition of active chromatin characteristics on day four and a transition to a repressive state on day 14, and identified distinct transcription factor ensembles associated with these events. The rapid transcriptional response involves the engagement of super-enhancers at genes implicated in extracellular matrix reorganization and TOR signaling, while H3K4me3 breadth highly correlates with transcriptional activity and dynamic changes at genes involved in proteolysis, cell cycle activity, and cell differentiation. Using loss- and gain-of-function approaches, we identified transcription factors in cardiomyocytes and endothelial cells influencing cardiomyocyte dedifferentiation or proliferation. Finally, we detected significant evolutionary conservation between regulatory regions that drive zebrafish and neonatal mouse heart regeneration, suggesting that reactivating transcriptional and epigenetic networks converging on these regulatory elements might unlock the regenerative potential of adult human hearts.

## Graphical Abstract

**Graphical Abstract:**
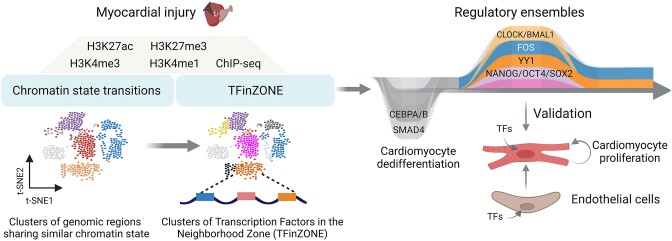

## Introduction

Heart failure is the leading cause of death globally, and the limited regenerative capacity of the human heart significantly contributes to high morbidity and mortality. In adult mammalian hearts, cardiomyocytes (CMs) fail to re-enter the cell cycle and to proliferate following heart injury (1–3). In contrast, adult zebrafish and neonatal mouse CMs can divide, leading to complete heart regeneration following heart injury (4–6). While neonatal mouse hearts' ability to regenerate might be due to their proliferative state in a short developmental window (until postnatal day 7, i.e. P7) (5,7,8), adult zebrafish CMs can regenerate the heart upon injury through CM cell cycle re-entry. Zebrafish heart regeneration involves a close interaction between two key components: CMs, which act as the primary source of cells in the regenerating heart through dedifferentiation, proliferation, and signaling, and non-muscle cells (9–12). The puzzling question is why adult mammalian CMs fail to reactivate transcriptional programs supporting heart regeneration following injury. In the last decade, many studies have reported an increase in adult CM proliferation rates through the use of cell cycle regulators, transcription factors (TFs), microRNAs as well as growth factors and their receptors (13–23). However, this increase is marginal. To unlock the full potential of CMs to proliferate upon injury and efficiently regenerate the heart, we need to better understand how other model organisms, such as zebrafish, can overcome the intrinsic epigenetic barrier and reactivate transcriptional networks that drive regeneration. This understanding can provide essential insights into how regeneration is achieved or could potentially be stimulated in adult human hearts.

Distinct transcriptional states of identical genomes lead to diverse cellular phenotypes and properties, which is achieved through epigenetic regulatory mechanisms. Epigenetic regulation plays a crucial role in instructing cell proliferation, fate determination, and cell plasticity (24–27). Histone modifications are particularly important and can correlate with active, repressed, and poised gene expression states that define cellular function and response to signaling cues (26,28–30). While much knowledge has been gained about the transcriptional changes associated with zebrafish heart regeneration, little is known about how chromatin states change during the regenerative process. Most studies so far have focused on single histone marks at a specific time point of zebrafish heart regeneration. For example, ChIP-sequencing (seq) for acetylated lysine 27 of histone H3 (H3K27ac) at 7 days after tamoxifen-inducible genetic ablation of around half of CMs in the zebrafish heart identified small regulatory elements, known as tissue regeneration enhancer elements (TREEs), that direct the regenerative transcriptional response upon injury (31). A follow-up study involving ChIP-seq of H3.3 and H3K27ac at 14 days after CM ablation identified many *cis-*regulatory sequences marked by H3.3 and H3K27ac occupancy and predicted TF that might be involved in heart regeneration (32). Another study using apical resection showed a strong correlation between reduced expression of genes encoding sarcomere and cytoskeletal proteins and increased H3K27me3 within their loci following cardiac injury, which was required for zebrafish heart regeneration (33).

In this study, we present a comprehensive analysis of the histone modifications H3K4me3, H3K4me1, H3K27me3 and H3K27ac across multiple time points during zebrafish heart regeneration. Our analysis reveals changes in the chromatin states that occur throughout the regenerative process and provides insights into the regulatory networks that control cardiac regeneration. This information can potentially be used to develop regenerative therapies for heart disease.

## Materials and methods

### Animal studies

All animal experiments were performed according to the regulations issued by the Committee for Animal Rights Protection of the State of Baden-Württemberg (Regierungspraesidium Karlsruhe).

### Zebrafish cryoinjury

Zebrafish care, breeding and cryoinjury experiments on 3–6-month old fish of the same genetic background were performed as described previously (34,35).

### Cell lines and cell culture

HEK293T cells were purchased from ATCC (CRL-3216) and cultured in DMEM supplemented with 10% fetal bovine serum (FBS, Thermo Fisher Scientific, 10270106), 1% penicillin–streptomycin (Thermo Fisher Scientific, 15140122) and 2 mM l-glutamine (Thermo Fisher Scientific, 25030123).

Human umbilical vein endothelial cell (HUVECs) were cultured in Endothelial Cell Growth Medium MV 2 (PromoCell, C-22022), supplemented with 1% penicillin–streptomycin.

Neonatal CMs were isolated from 4-day-old (P4) mice using a neonatal heart dissociation kit (Miltenyi Biotec, 130–098-373) and neonatal mouse CM isolation kit (Miltenyi Biotec, 130-100-825), according to the manufacturer's instructions. The cells were cultured on coverslips in 24-well plates or dishes pre-coated with 10 μg/ml Laminin (Corning, 354232) in DMEM/F12 medium supplemented with 1% l-glutamine, 1% Na-pyruvate,1% non-essential amino acids, 1% penicillin/streptomycin, 5% horse serum and 10% FBS.

### siRNA-mediated silencing in neonatal cardiomyocytes

10^5^ freshly isolated P4 CMs were seeded on coverslips in a 24-well plate pre-coated with 10 μg/ml Laminin. 24 h later, the medium was exchanged to a fresh medium, and cells were transfected with either 50 nM scrambled siRNA (Dharmacon/GE, D-001810-10-05) or 50 nM siRNA against *Clock* (Dharmacon/GE, L-040484-01-0005), *Bmal1* (Dharmacon/GE, L-040483-01-0005), *Cebpa* (Dharmacon/GE, L-040561-00-0005), *Cebpb* (Dharmacon/GE, L-043110–00-0005), *Yy1* (Dharmacon/GE, L-050273-00-0005), *Hand2* (Dharmacon/GE, L-057649-01-0005), *Smad4* (Dharmacon/GE, L-040687-00-0005) using Lipofectamine RNAiMax (Thermo Fisher Scientific, 13778–075). 48 h after transfection, CMs were subjected to further analysis, i.e. FACS, qPCR and immunostaining.

### Lentivirus-mediated overexpression in neonatal mouse cardiomyocytes and HUVECs

HEK293T cells were seeded on a 6-well plate in DMEM, high glucose, GlutaMAX (Gibco, 61965059) supplemented with 10% fetal bovine serum and 1% penicillin-streptomycin. At 70% confluence, HEK293T cells were transfected with 1.5 μg OCT4 (TRCN0000475990), SOX2 (TRCN0000473508), NANOG (TRCN0000474074), GATA2 (TRCN0000480188), FOS (TRCN0000488098), YY1 (TRCN0000480857), BMAL1 (TRCN0000481576) lentiviral constructs obtained from the RNAi consortium (TRC) overexpression library along with 0.975 μg CMVΔR8.74 packaging plasmid and 0.525 μg VGV.G envelope plasmids using the X-tremeGENE DNA transfection reagent (Roche, 6366236001). 48 h after transfection, the viral supernatant was collected and used to transduce neonatal CMs or HUVECs in the presence of 8 μg/ml polybrene (Sigma, TR-1003-G). 48 h after transduction, CMs were subjected to further analysis while HUVECs were selected with 10 ng/mL puromycin for 48 h and used for co-culture with neonatal mouse CMs.

### Co-culture of HUVECs with neonatal mouse cardiomyocytes

10^5^ HUVECs overexpressing the TFs of interest were seeded on a 6-well plate. Twenty-four hours later, 10^5^ freshly isolated P4 mouse CMs were seeded on the top of the HUVECs and cultured in a 1:1 mix of CM medium (DMEM/F12 medium supplemented with 1% l-glutamine, 1% Na-pyruvate,1% non-essential amino acids, 1% penicillin/streptomycin, 5% horse serum and 10% FBS) and EC medium (EGM-MV2 supplemented with 1% penicillin/streptomycin) for 48 hours.

### EdU incorporation to assess cell proliferation

Cells were labelled with 5-ethynyl-2′-deoxyuridine (EdU) for 2 h and staining was performed using with Click-iT™ Plus EdU Alexa Fluor™ 647 Flow Cytometry Assay Kit (ThermoFischer, C10634) according to the manufacturer’s instructions.

### Immunofluorescence staining

Cells were permeabilized with 0.5% Triton X-100 in PBS for 10 min. After blocking in 3% BSA in PBS for 30 min, cells were incubated with anti-phospho-histone H3 (Ser10) (Millipore, 06-570, 1:100) and anti-α-actinin (Boster Biological Technology, MA1104, 1:100) antibodies in 0.3% Triton X-100/ 1% BSA/ 1xPBS overnight in a humidified chamber at 4°C. After three consecutive five minutes washes in PBS, cells were incubated for another hour with a corresponding secondary antibody, conjugated to Alexa 555 or Alexa 488 in PBS followed by DAPI staining.

### RNA isolation, RT-PCR and real-time PCR

Total RNA was isolated with TRIzol RNA Isolation Reagent (Invitrogen, 15596018). For real-time PCR analysis cDNA was synthesized with the High Capacity cDNA Reverse Transcription Kit (Applied Biosystems, 4368813) and real-time PCR was performed using qPCRBIO SyGreen Blue Mix (Nippon, PB20.16-51). Cycle numbers were normalized to these of α-Tubulin (Tuba1a).

### ChIP-sequencing

For each experimental repetition, three zebrafish hearts were fixed in 1% formaldehyde at room temperature for 10 min. To stop the formaldehyde fixation, the hearts were then treated with 125 mM glycine while shaking on ice for 10 min. After three times washes with ice-cold PBS for 10 minutes each, the hearts were lysed in 200 μl of ice-cold L1 lysis buffer (50 mM Tris pH 8.0, 2mM EDTA pH 8.0, 0.1% NP40, 10% glycerol), supplemented with 1× protease and 1x phosphatase inhibitors, and left on ice for 10 min. The lysate was then centrifuged, and the pellet was resuspended in L2 buffer (1% SDS, 5 mM EDTA pH 8.0 and 50 mM Tris pH 8.0), also containing protease and phosphatase inhibitors, and sonicated using a tip sonicator three times for 10 s, followed by chromatin shearing using Covaris (Chromatin shearing settings) for 25 min. The chromatin was diluted 1:10 with DB buffer (0.5% NP40, 200 mM NaCl, 5 mM EDTA pH 8.0, 50 mM Tris pH 8.0), supplemented with proteinase and phosphatase inhibitors. To remove nonspecific binding, preclearing was performed by incubating the lysate with 80 μl of protein G Sepharose 4 fast flow beads (GE Healthcare 17-0618-01) for 3 h at 4°C with rotation. Each experimental repetition was divided into four samples, and each sample was subjected to immunoprecipitation using specific antibodies for H3K4me3 (ab8580), H3K4me1 (ab8895), H3K27me3 (Millipore 07449) or H3K37ac (ab4729). For IP, 0.5 μg of the corresponding antibody was added to the lysate and incubated overnight at 4°C with slow rotation. After incubation, 50 μl of protein G Sepharose 4 fast flow beads (GE Healthcare 17-0618-01) per milliliter of lysate were added to each tube, followed by gentle rotation at 4°C for 1 h. The samples were then centrifuged for 30 s, and the supernatant was discarded. The beads were washed three times with NaCl buffer (SDS 0.1%, 1% NP40, 2 mM EDTA pH 8.0, 500 mM NaCl, 20 mM Tris pH 8.0), followed by washes with LiCl buffer (SDS 0.1%, 1% NP40, 2 mM EDTA pH 8.0, 500 mM LiCl, 20 mM Tris pH 8.0), and finally with TE buffer (Tris pH 8 20 mM, EDTA 2 mM). The chromatin was then eluted from the beads using water. To reverse the cross-linking, the eluted chromatin was incubated at 65°C overnight. DNA was subsequently extracted using a phenol/chloroform extraction method, and the concentration of DNA was measured using a Pico green kit. Library preparation and sequencing were performed on a NextSeq500 (Illumina) platform.

### Data analysis

All raw reads were trimmed using Trimmomatic-0.36 (36) with the parameters (ILLUMINACLIP:<ADAPTERS>:2:30:10 LEADING:3 TRAILING:3 SLIDINGWINDOW:4:15 MINLEN:20 CROP:70 HEADCROP:10. The trimmed reads were mapped using Bowtie2 (v.2.4.4) (default settings) to the zebrafish genome (danRer11). The SAM output files from the mapping program were converted to BAM format and sorted using the samtools view command with the ‘-Sb’ option. Next, PCR duplicates were removed from the BAM files using the MarkDuplicates.jar tool from Picard (version 1.119). The BAM files were then merged using the bamtools merge command, and from the merged files, bigwig files were created using bamCoverage from deeptools (37), with the following parameters: (-bs 20 –smoothLength 40 -p max –normalizeUsing RPKM -e 150).

To identify peaks, we used macs2 (version 2.1.4) with selected settings that best correlated with the visualization of the ChIP-seq reads in the genome browser. For H3K27me3, the following settings were used: (–broad -g 1.4e + 9 -q 0.01 –keep-dup 1 –fix-bimodal –nomodel –extsize 520). For the H3K4me3 and H3K27Ac, the settings were similar to H3K27me3 but with ‘–extsize 200″. Peaks were annotated using annotatePeaks.pl with danRer11 genome and a custom General Feature Format (GTF) file that combined genes from ENSEMBL, REFSEQ, and NONCODE version 5. The NONCODE data from the NONCODE project (http://www.noncode.org/download.php) was liftovered from danRer10 to danRer11 using the liftOver script from UCSC. To quantify the peaks and calculate differential peak enrichment, all peaks from different time points were merged using the concatenate function from bash (cat), sorted, and merged using bedtools merge (-d 100). Peaks were annotated into three main regions: promoter (peaks located within TSS ± 2 kb), genebody (peaks > 2 kb from TSS and overlapping exons, introns, and 3′-UTR), and intergenic (peaks located outside these regions) using a R script. The number of genes marked by H3K4me3 was determined using a custom R script (03aH3K4me3_DENSITY_genes.Rmd) that cross-referenced the gene names from the ENSEMBL database with the annotated peaks from the peak list. The percentage of marked and unmarked genes was plotted using geom_area from ggplot2 (version 3.4.0) in R.

To calculate the breadth of H3K4me3 peaks, we first performed summary statistics of the peak size from the merged peak list. If the size of a peak was equal to or higher than the median quantile (Q2) (top 50% of peak wide) it was considered as a broad peak, if the peak was lower than Q2 (bottom 50% of peak wide) it was considered as a narrow peak.

After calling of H3K27ac peaks, Rank Ordering of Super-Enhancer (ROSE) (38) was utilized to identify super-enhancers (SE) and typical enhancers (TYE) using default settings.

### Correlative analysis between histone modifications and gene expression

Correlation between the histone modification and the RNA expression was performed by Spearman's rank correlation using the function cor.test from the library stats in R. Genes annotated to the histone mark peaks were intersected with RNA-seq gene expression data from (35).

Matrices of *z*-scores for the log_2_ of the mean reads per kilobase of mapped reads (RPKM) count from both datasets were created. Bedtools (multicov -bams -bed) (v2.30.0) was used to calculate the enrichment of histone marks on the merged peaks, while the analyzeRepeats.pl function (Ensembel_data danRer11 -strand both -count genes) was utilized to count raw mapped reads from the RNA-seq datasets. RPKM were calculated with the function rpkm.default from edgeR (v.3.36.0) (default settings).

### K-means clustering based on epigenetic signatures

For cluster analysis of histone marks, we used the normalized matrix of RPKM counts (*z*-score of the log_2_ of RPKM of the count). Within groups sum of squares versus the number of clusters was used to determine the optimal number of clusters for further analysis. The *k*-means function from stats (settings 6, iter.max = 500, algorithm =‘Hartigan-Wong’) was used to perform unsupervised cluster analysis. For H3K27ac, we used only peaks positively correlating with gene expression. For H3K4me3, we filtered broad and narrow-sized peaks with either a positive or negative correlation to the expression of the annotated gene. A gene was considered correlated if, after the Spearman's rank correlation test from the cor.test function, the *P*-value was lower in the bottom 25% of the value, i.e. negative correlation rho ≤ −0.3; positive correlation rho ≥ 0.3. We compared the correlation of normalized counts (*z*-score of log_2_ from meanRPKM + 1) from RNA-seq and normalized enrichment of histone marks during the time points from 0 to 45 dpci.

### Classification of the different types of *cis-*regulatory areas

In order to classify the different chromatin signatures during zebrafish heart regeneration, we merged H3K4me1, H3K4me3, H3K27me3, and H3K27ac ChIP-seq peaks. Quantification was performed with the help of bedtools (multicov -bams -bed) (v2.30.0) (default settings) and the normalized RPKM +1 values were converted in log_2_ values, from which *z*-score of each histone enrichment was calculated. To simplify the analysis, we considered a genomic area to be active if markers such as H3K4me1 and H3K27ac accumulation passed their quartile 1 (Q1). If the value was lower than Q1, we considered the area unmarked. For H3K4me3, border value was 1. Repressed areas were identified as those having H3K27me3 ≥ Q1, H3K27ac ≤Q1, and H3K4me3 ≤ 1. We further classified the functional elements into five groups, based on whether they changed from active to repressed chromatin state or vice versa when compared to a reference time point. The groups were named A_Ia, Ia_A, U_Ea, U_Ia and U_Pa, and were composed of various active and inactive epigenetic signatures presented in Figure 3A. To represent the dynamics of the divergent areas, we created an alluvial plot using the geom_alluvium function with settings (aes(fill = divergent_Iaegions), curve_type = ‘arctangent’).

### Gene ontology

The gene ontology (GO) analysis in the manuscript was performed using the compareCluster function from clusterProfiler (version 4.2.2) with the following settings: fun =‘enrichGO’,OrgDb=‘org.Dr.eg.db’, ont=‘BP’,pAdjustMethod =‘BH’,pvalueCutoff = 0.05, qvalueCutoff = 0.05, readable = T. The representative GO terms in each group were selected based on the output matrix with a significant *P*-value of <0.05 after the over-representation analysis (ORA) test and containing genes unique to the list of genes.

### Motifs and transcription factor networks discovery—TFinZONE

**Figure 1.**
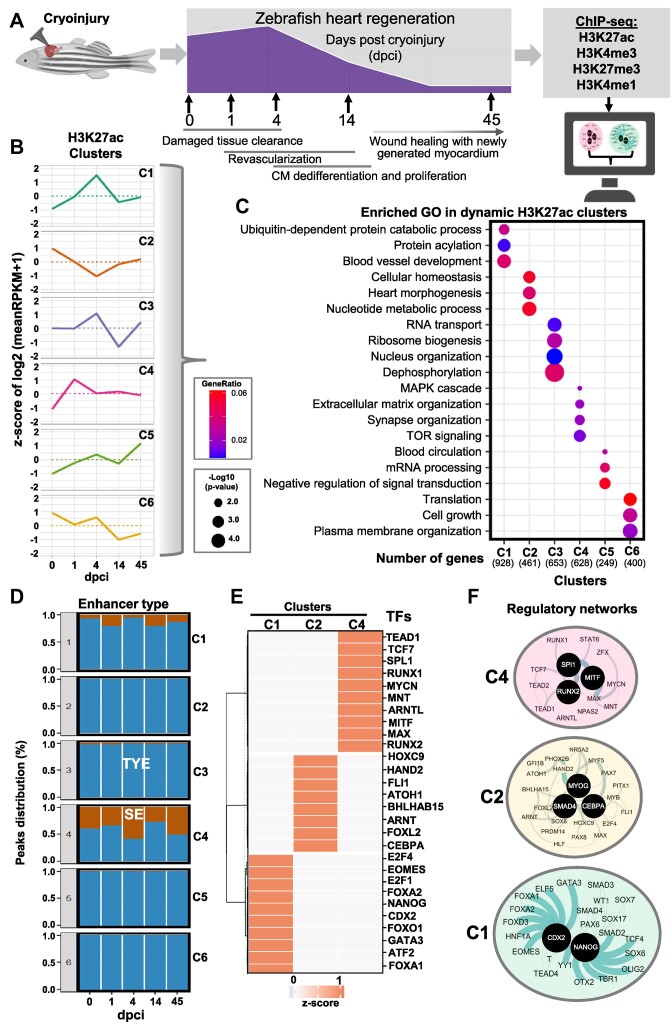
H3K27ac dynamics during zebrafish heart regeneration. (**A**) Schematic representation of the experimental setup of the study. (**B**) Unsupervised k-means clustering of normalized enrichment of H3K27ac at different time points after cryoinjury of zebrafish hearts. Clustering was performed using positively correlating H3K27ac peaks with the expression of associated genes. Values are the *z*-score from the log2 of the mean of RPKM + 1. (**C**) Dot plots displaying the representative gene ontology (GO) terms of genes associated to peaks in the different H3K27ac clusters. (**D**) Percentage of super enhancers (SE, brown) and typical enhancers (TYE, blue) in the different clusters during the course of regeneration. Rank Ordering of Super Enhancer (ROSE) for H3K27ac peaks was performed to identify SE. (**E**) Heatmap showing the TF motifs associated to the different clusters of H3K27ac. (**F**) Interaction network of TFs identified by motif analysis of genomic regions within clusters 4 (C4) (top panel), cluster 2 (C2, middle panel) and cluster 1 (C1, bottom panel). For this and the following figures, black circles represent TFs with the highest interaction score according to STRING database (default settings). The thickness of the line connecting the different proteins represents the strength of the protein-protein interaction.

**Figure 2.**
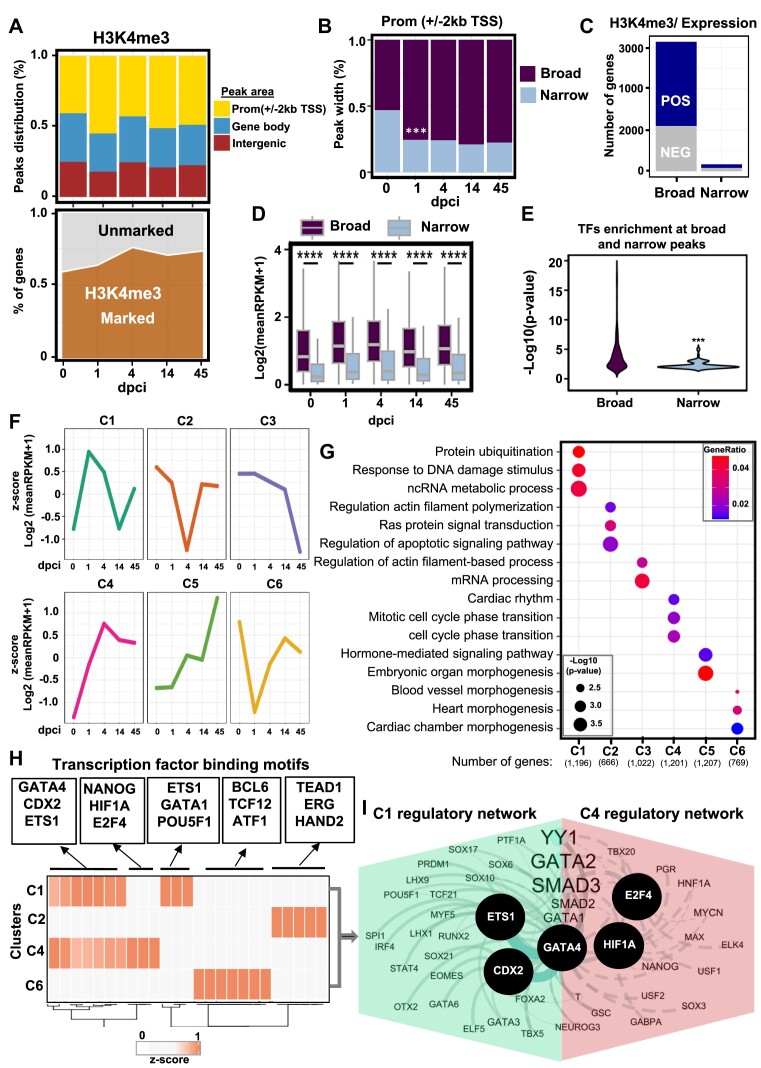
H3K4me3 breadth correlates with transcriptional activity in zebrafish heart regeneration. (**A**) Distribution of H3K4me3 peaks at the promoter (TSS ± 2 kb) (yellow), genebody (TSS > 2 kb) (blue), and intergenic regions (maroon) (top panel). Values are the percentage of each region at each time point. Density plot showing the percentage of genes associated with H3K4me3 peaks (bottom panel). Values are the percentage of marked genes (dark brown) with at least one peak of H3K4me3. (**B**) Stacked bar plot showing the breadth of H3K4me3 peaks at different time points after heart injury. Broad peaks (>0.5 kb; top 50%) (dark violet), and narrow peaks (<0.5 kb; bottom 50%) (light blue). *P*-values **P < 0.001 calculated after Fisher's exact test. (**C**) Percentage of genes characterized by broad and narrow H3K4me3 peaks, positively or negatively correlating to the expression of the neighboring gene, after Spearman's rank correlation**. (D**) Boxplot showing the expression of genes associated with H3K4me3 peaks, separated by the broadness of the H3K4me3 peak. Values are the log2 from the mean of reads per kilobase million (RPKM)+1. (**E**) Violin plot showing the transformed log10 from the p-value after hypergeometric test of findMotifsGenome.pl from Homer over the background. (**F**) Unsupervised k-means clustering of the normalized enrichment of H3K4me3 at broad peaks correlating with gene expression. Correlation after Spearman′s rank test correlation (rho ≤ -0.3 or rho ≥ 0.3 and *P*-value < 0.35). Values are the *z*-score from the log_2_ of the mean of RPKM + 1. (**G**) Dot plots displaying the representative gene ontology (GO) terms of genes associated with H3K4me3 peaks in the different clusters. *P*-value was determined by enrichGO from clusterprofiler after ORA test. (**H**) Heatmap showing enriched TF motifs in the selected clusters. The associated factors were selected according to the workflow presented in Supplementary Figure S1C. The values represent the *z*-score of the –log_10_*P*-value. (**I**) Interaction network of TFs enriched in C1 (green) and C4 (pink).

To identify both known and novel motifs from the peaks, we used the findMotifsGenome.pl tool from homer (v.3.12) with the settings (-size 500 -len 8 -h -mask) and performed a hypergeometric test. To determine if the known motifs are found within the same ChIP-seq peaks, we developed a custom R script to assess whether motifs were found together at specific genomic areas. We combined the peaks from the different divergent areas and the identified motifs with the help of the function findMotifsGenome.pl with the settings (peaks.bed danRer11 output_folder -size 500 -len 8 -h -mask -p 16 -cache 3000 -find known_motif_matrix.txt). To visualize genomic regions enriched for common TF motifs, we used the Seurat (v.4.1.1) (39) pipeline with the functions FindNeighbors (dims = 1:10), and FindClusters (resolution = 0.5, random.seed = 1, algorithm = 1). UMAP with the function RunUMAP (dims = 1:30, n.neighbors = 30) and RunTSNE (Seurat_ object, dims = 1:20, check_duplicates = FALSE) was used to plot the dimensionality reduction. We identified significantly enriched TFs per chromatin state transition clusters using the FindAllMarkers settings (only.pos = TRUE, min.pct = 0.10, logfc.threshold = 0.09, test.use=‘bimod’). The list of TFs enriched in each cluster was loaded into STRING (v.11.5) (40) to identify protein-protein interactions. We imported the output from STRING into a custom R script (01Fig1E_MO_H2K27ac_aSTRING.Rmd for H3K27ac and 02Fig2H_MO_H3K4me3_aSTRING.Rmd for H3K4me3 related networks) to identify the TFs involved in highest number of protein interactions. Finally, Cytoscape (v.3.6.1) (41) was used to create a custom network.

For motifs analysis in conserved regions, first we used Liftover web-base program (default settings) to convert the genomic regions showing dynamic chromatin state transitions at 4 dpci in zebrafish to mouse genome coordinates (mm10 version). Motifs search in the conserved regulatory sequences was performed using findMotifsGenome.pl tool from Homer (v.3.12) with the above settings.

### ChIP-seq and RNA-seq data visualization

The interactive genome browser (IGV) (v.2.13.2) program was used to visualize normalized bigwig files from the ChIP-seq data, as well as the peak files.

### Published data used in this study

RNA-seq data at different time points of zebrafish heart regeneration has been deposited in PRJNA509429 repository (35). Single-cell from neonatal hearts (GSE153481), CM single-cell data (GSE130699) and ChIP-seq from H3K27ac (GSE123868) were retrieved from previously published studies.

### Code availability

Codes have been deposited in GitHub (https://github.com/jcorder316/01TFinZONE) and are also available upon request to the corresponding authors.

## Results

### The rapid transcriptional response of the heart to injury involves the engagement of super-enhancers

To identify epigenetic signatures and transcriptional networks driving cardiac regeneration, we investigated the dynamics of chromatin states after inducing myocardial necrosis in adult zebrafish through cryoinjury (34,35). The dynamic epigenetic response was detected at days 1, 4, 14 and 45 after cryoinjury (1, 4, 14, 45 dpci) using chromatin immunoprecipitation and massive parallel sequencing (ChIP-seq) for different histone modifications including: H3K4me1, H3K4me3, H3K27me3 and H3K27ac (42) (Figure 1A, Supplementary Figure S1A). These time points were selected based on our previous study showing major transcriptional dynamics at these stages (35).

Since H3K27ac is present at active promoters and enhancers and highly correlates with gene activation, we first performed unsupervised cluster analysis of H3K27ac changes correlating with gene expression changes during the course of cardiac regeneration (35). A total of 2102 H3K27ac changes correlated with changes in gene expression (Supplementary Figure S1B and S1C). These genes fell into different clusters (Figure 1B, and Supplementary Table S1). Cluster 4 (C4) showed a rapid increase of H3K27ac already at 1 dpci which decreased at later time points, while cluster 1 (C1) was characterized by a gradual increase in H3K27ac with a peak at 4 dpci and a decrease at later time points. In contrast cluster 2 (C2) showed the lowest level of H3K27ac at 4 dpci. GO analysis revealed that C4 was enriched for genes involved in MAPK and TOR signaling, and extracellular matrix (ECM) organization (Figure 1B, C). Genes that were characterized with highest level of H3K27ac at 4 dpci were involved in blood vessel development, ubiquitin-mediated catabolic process and protein acylation. Genes in C2, which showed a decrease in H3K27ac at 4 dpci were associated with cellular homeostasis, heart morphogenesis, and nucleotide metabolic processes. C6 which showed high dynamics, i.e. a decrease at 1 dpci, an increase at 4 dpci followed by a decrease at 14 dpci, was mainly enriched for genes involved in cell growth, plasma membrane organization, and translation, while C5, which was characterized by a major increase in H3K27ac at 45 dpci, contained genes involved in negative regulation of signal transduction, blood circulation, and mRNA processing (Figure 1C).

H3K27ac is a signature of both super-enhancers (SE) and typical enhancers (TYE). SE is a small set of enhancers spanning large genomic regions, which have been reported to control the expression of cellular identity genes (43). We next applied the ROSE algorithm to distinguish between SE and TYE in the dynamic H3K27ac clusters. Interestingly, C4 which showed a peak of H3K27ac at 1 dpci, showed a high percentage of H3K27ac peaks associated with SE areas, suggesting that the rapid transcriptional response involves the engagement super-enhancers. Other clusters were mainly enriched in TYE (Figure 1D). To identify upstream regulatory factors that drive transcriptional responses during cardiac regeneration we performed motif enrichment and protein network analysis (Supplementary Figure S1D). C4, which was associated with the rapid transcriptional response involving engagement of super-enhancers, was enriched for motifs for TFs that activate expression of cell cycle regulators, e.g. MYCN (44), MITF (45) and TEAD-s, (17,46), ARNTL (BMAL1) among others (Figure 1E). Network analysis revealed that these factors build protein-protein interaction networks around MAX/MYCN, MITF, SPI1 and RUNX TFs (Figure 1F). Cluster C1, which showed a peak at 4 dpci, was enriched for motifs for TFs that have been shown to regulate heart growth and regeneration, such as FOX, GATA-s, SOX, and central players within this network were CDX2 and NANOG (Figure 1E, F). C2, which showed opposite dynamics, i.e. decreased H3K27ac at 4 dpci, was enriched for SMAD4, HAND2, PHOX2B, MYOG and CCAAT Enhancer Binding Protein Alpha (CEBPA), suggesting that CM dedifferentiation and proliferation might be stimulated by lowering the activity of these TF assemblies (Figure 1E, F). In our analysis, we identified three clusters that showed major dynamics throughout the different time points (Supplementary Figures S2A, S2B). Cluster C3, which includes genes related to RNA transport, ribosome biogenesis, and nuclear organization, showed enrichment for GATA4, CEBPA, ETS1 and various FOX TF motifs. Meanwhile, Cluster C5, consisting of genes involved in mRNA processing and signal transduction, exhibited enrichment for TFs implicated in the regulation of circadian rhythm and cell differentiation, including BMAL1, CLOCK, BHLHE41 and MYOD1. Additionally, Cluster C6, composed of genes linked to translation, cell growth, and plasma membrane organization, demonstrated enrichment in FOS, E2F4 and IRF4 motifs (Supplementary Figures S2A, S2B).

### Increased breadth of H3K4me3 domains correlates with robust transcriptional activity and dynamics

The trimethylation of histone H3 lysine 4 (H3K4me3) marks active gene promoters (42,47,48). Large H3K4me3 domains spread over genes are essential for cell identity and function as well as to ensure their transcription precision (47). Therefore, we analyzed the H3K4me3 dynamics in more detail. We identified around 40000 peaks of H3K4me3 per time point, with the highest number of peaks at 4 dpci (Figure 2A, bottom, Supplementary Figure S3A). That aligns with the observed highest number of upregulated genes at 4 dpci compared to other days (35). Already at 1 dpci there was an increase in H3K4me3 at promoters (Figure 2A). Interestingly, the breadth of H3K4me3 domains also increased at 1 dpci compared to day 0 and was kept until 45 dpci (Figure 2B, Supplementary Figure S3B). We next assessed if the broadness of H3K4me3 domains correlates with the expression levels of the associated genes during zebrafish heart regeneration. Importantly, broad peaks highly correlated with expression levels, while narrow peaks did not show major correlation (Figure 2C). H3K4me3 marks also poised promoters, which in addition to the H3K4me3 mark contain an H3K27me3 mark (49), providing an explanation to the observed both positive or negative correlation of broad H3K4me3 peaks with expression level. Intersecting H3K4me3 peaks with different breadth with RNA-seq data revealed that broad H3K4me3 domains at the promoter region were associated with higher transcription levels compared to narrow peaks and contained significantly higher binding sites for regulatory factors (Figure 2D, E, Supplementary Figure S3C). Given the significant correlation only of broad H3K4me3 with expression, we next performed an unsupervised cluster analysis of broad H3K4me3 peaks which either positively or negatively correlated to the expression of the associated genes (Figure 2F). The analysis identified six clusters (Figure 2F, Supplementary Figure S3D). C1 and C4 showed significantly enrichment of H3K4me3 either at day 4 or 1 after cryoinjury, respectively, and were highly positively correlated with gene expression (Figure 2F, Supplementary Figure S3E). C2 and C6, showed a significant depletion of H3K4me3 at 4 and 1 dpci, respectively, and recovered by 14 dpci. Interestingly, the decrease in H3K4me3 in these clusters negatively correlated with gene expression. C3 and C5 either progressively lost or gained H3K4me3 only at 45 dpci (Figure 2F, Supplementary Figure S3E, Supplementary Table S2). GO analysis revealed that cardiac injury-activated cluster C1 was enriched in genes associated with protein ubiquitination, cellular response to DNA damage stimulus, and ncRNA metabolic process, while C4 was enriched in circadian rhythm, and mitotic cell cycle phase transition (Figure 2F, G). The C2, which showed decrease in H3K4me3 at 4 dpci, was enriched in genes associated with negative regulation of actin filament polymerization, Ras protein signal transduction, and regulation of apoptosis signaling pathway, while C6 cluster was enriched in blood vessel formation, heart morphogenesis, and cardiac chamber morphogenesis (Figure 2F, G). For clusters C3 and C5 the associated GOs were mainly connected to actin filament organization and hormone-mediated signaling pathway (Figure 2F, G). Motif analysis within peaks of genes in clusters associated with increase of H3K4me3 (C1 and C4) identified significant enrichment of GATA4, CDX2 and ETS1 motifs in both clusters, while enrichment of NANOG, HIF1A and E2F4 was specific for C4 (Figure 2H). Protein network identification of significantly enriched TFs in C1 and C4 identified ETS1, CDX2, GATA4, HIF1A, E2F4 as core factors associated with H3K4me3 increase and transcriptional activation (Figure 2H, I). Clusters characterized by decreased H3K4me3 at 1 dpci were enriched in motifs for BCL6, ATF1 and TCF12 among others, while clusters characterized by decreased H3K4me3 at 4 dpci were enriched for TEAD1, ERG and HAND2 (Figure 2H).

We next examined whether a similar pattern could be detected in H3K27me3 peaks. Most of the H3K27me3 marked genomic regions were found outside of the TSS (around 75% of the identified peaks), suggesting a role for H3K27me3 in the repression of distal regulatory elements in cardiac homeostasis (Supplementary Figure S3F). Interestingly, already 1 day after injury there was a major shift of H3K27me3 to promoters, supporting the important role of H3K27me3-mediated silencing during regeneration (33) (Supplementary Figure S3F). In line with the large-scale gene activation upon injury (35), H3K27me3 marked genes showed an opposite pattern to H3K4me3, e.g. H3K27me3 marked genes were reduced at the early days of heart regeneration (Supplementary Figures S3F, S3G). Unlike H3K4me3, there was no significant change in the broadness of H3K27me3 domains (Supplementary Figure S3H).

### Regulatory ensembles associated with chromatin state dynamics in heart regeneration

**Figure 3.**
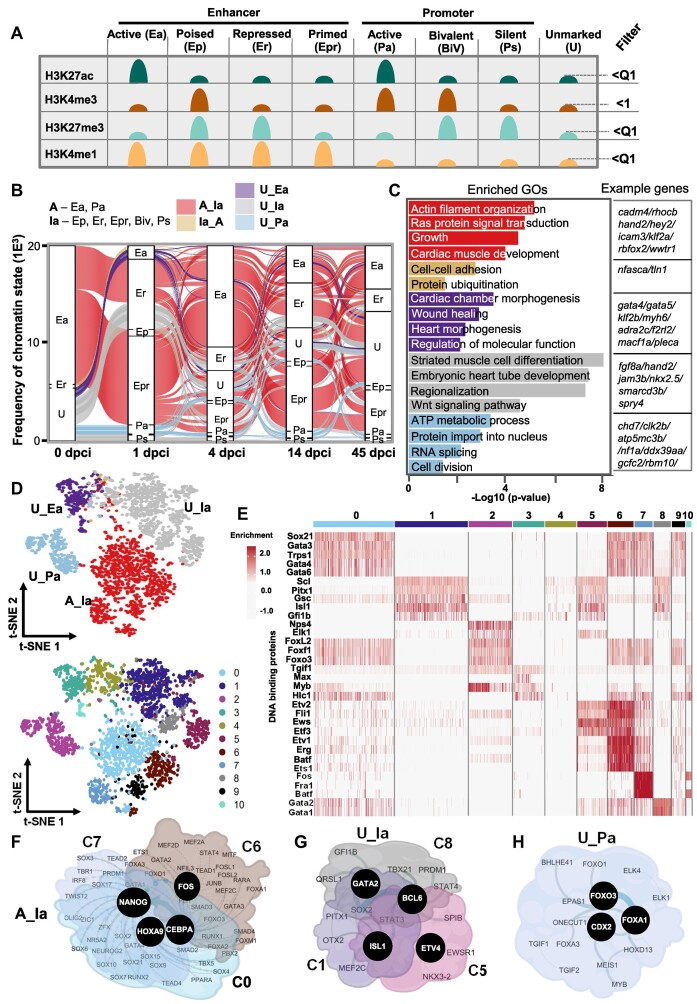
Large-scale acquisition of repressive chromatin marks one day after heart injury. (**A**) Schematic representation of the different chromatin states. (**B**) Alluvial plot representing the dynamic of chromatin states in (A) with reference point 1 dpci. Activated enhancers and promoter areas (A) changing from active at 0 dpci to inactive (Ia) at 1 dpci (A_Ia, red). Inactive areas were the combination of Ep, Er, Epr, BiV and Ps. Transitions from inactive to active chromatin state at 1 dpci (Ia_A, light brown), unmarked areas to active enhancers (U_Ea, violet), unmarked areas to inactive chromatin states (U_Ia, grey), unmarked areas to active promoters (U_Pa, light blue). The Y-axis values are frequency of the assigned divergent region. Unmarked, represents genomic area without any significant enrichment of the studied histone marks, i.e. bellow the Quartile 1 (Q1) for H3K27ac, H3K27me3 and H3K4me1, and value of 1 for H3K4me3. (**C**) GO terms of the dynamic chromatin state groups presented in panels A and B. Example genes from each GO are presented to the right. (**D**) T-distributed stochastic neighbor embedding (t-SNE) visualization displaying the dynamic chromatin state transition groups (top) and the distribution of TF motifs (bottom). (**E**) Heatmap showing the enrichment of TF motifs in the clusters presented in (D, bottom panel). (F–H) Interaction network of TFs with enriched motifs in clusters associated with A_Ia transition (**F**), in clusters associated with U_Ia transition (**G**) and C2 associated with U_Pa transition (**H**).

**Figure 4.**
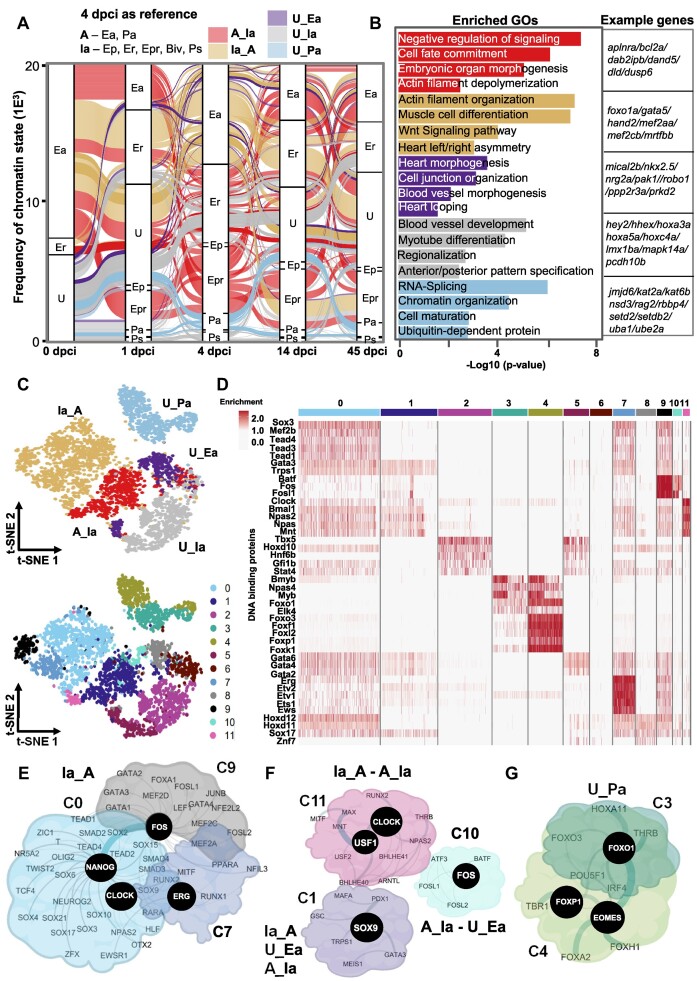
Switch between inactive to active chromatin states four days after injury. (**A**) Alluvial plot representing dynamic chromatin states with a reference point 4 dpci. (**B**) GO terms of the genes within the dynamic chromatin state groups presented in panel A. Example genes from each GO are presented to the right. The *P*-value was determined by ORA. (**C**) T-distributed stochastic neighbor embedding (t-SNE) visualization displaying the dynamic chromatin state transition groups (top) and the distribution of TF motifs (bottom). (**D**) Heatmap showing the enrichment of TF motifs in the clusters presented in (C, bottom panel). (E–G) Interaction network of TFs with enriched motifs in all clusters associated with Ia_A transition (**E**), the highly dynamic C1, C10 and C11 clusters (**F**) and C3 and C4 associated with U_Pa transition (**G**) at 4 dpci.

**Figure 5.**
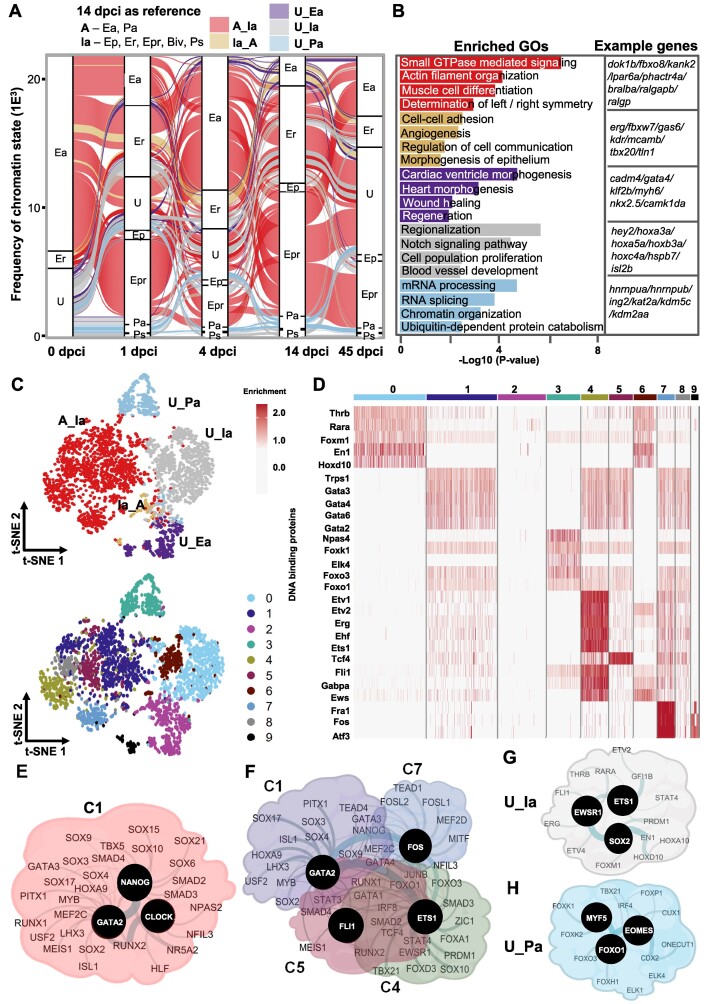
Transition from active to inactive chromatin state between 4 dpci and 14 dpci marks the start of the healing process. (**A**) Alluvial plot representing the dynamic chromatin states with reference point 14 dpci. (**B**) GO terms of the genes within the dynamic chromatin state groups presented in panel A. Example genes from each selected GO are presented to the right. The p-value was determined by enrichGO from clusterprofiler using ORA test; p-value *P < 0.05. (**C**) T-distributed stochastic neighbor embedding (t-SNE) visualization displaying the dynamic chromatin state transition groups (top) and the distribution of TF motifs (bottom). (**D**) Heatmap showing the enrichment of TF motifs in the clusters presented in (C, bottom panel). (E–H) Interaction network of TFs with enriched motifs in the largest cluster C1 associated with A_Ia transition (**E**), all clusters associated with A_Ia transition (**F**), with U_Ia transition (**G**) and cluster 3 associated with U_Pa transition (**H**) at 14 dpci.

H3K4me3 and H3K27ac are associated with active promoters and some highly active enhancers. H3K27me3 alone marks silent promoters, while in combination with H3K4me3 identifies bivalent (poised) promoters. H3K4me1 together with H3K27ac identifies active enhancers, H3K4me1 and H3K27me3 repressed enhancers, while a combination of these three marks identifies poised enhancers (Figure 3A). We next studied how these distinct chromatin states change during cardiac regeneration. Alluvial plots displayed highly dynamic chromatin states (Supplementary Figure S4). Therefore, we grouped the dynamics chromatin transitions in five groups: (1) group A_Ia: active enhancers and promoters, which switched to paused, primed, and repressed enhancers or bivalent and silent promoters and vice versa, i.e. Ia_A (2), unmarked chromatin regions that turned into active enhancers (3, U_Ea), active promoters (4, U_Pa) or acquired repressive characteristics (5, U_Ia). We next analyzed the chromatin state dynamics with reference time points 1 dpci, 4 dpci, and 14 dpci (Figures 3–5).

Using 1 dpci as a reference for the groups, we observed a major change from active to repressed chromatin states at 1 dpci, which at 4 dpci switched to active or repressed state, or lost histone marks (Figure 3B). This is consistent with the major increase in H3K27me3 at promoters 1 day after injury (Supplementary Figure S3F). Significant number of gene regulatory regions were unmarked before the injury but turned into active promoters, enhancers, or repressed promoters. GO analysis revealed that the group, which switched from active to repressed/poised chromatin state (A_Ia) at 1 dpci, was enriched for genes involved in Ras and small GTPase signaling transduction, growth, and actin filament organization (Figure 3B, C), in line with the decrease of H3K4me3 breadth at these genes (Figure 2F, G). The Ia_A group was enriched mainly for genes involved in protein polyubiquitination and cell–cell adhesion, consistent with the increase of H3K4me3 at these genes at 1 dpci (Figure 3C, Figure 2F, G). Unmarked chromatin regions which became active enhancers were associated with genes related to cardiac chamber morphogenesis, wound healing, and positive regulation of molecular function, while those that converted to active promoters were linked to ATP metabolic process and cell division, implying epigenetic priming of genes involved in cell cycling, metabolism and healing already at early stages of regeneration. Unmarked chromatin regions that gained repressive signatures (U_Ia) were linked to striated muscle cell differentiation, consistent with the important role of CM dedifferentiation in cardiac repair (Figure 3B, C). To identify the combination of TF associated with chromatin state transitions we utilized the Seurat pipeline. T-distributed stochastic neighbor embedding (t-SNE) in combination with uniform manifold approximation and projection (U-MAP), allowed us to visually represent the group of protein motifs identified within genomic regions and to assess their specificity for the distinct chromatin state groups. The hypothesis is that if factors are enriched within a cluster they might be controlling specific chromatin state transitions at a discrete subset of genes. Cluster analysis showed 11 distinct clusters (Figure 3D–H). C0, C6, C7 and C9 contained motifs within peaks specific for the A_Ia chromatin state transition group, suggesting that four distinct TF ensembles control the transition to inactive chromatin state (Figure 3D–F, Supplementary Table S3). The largest group C0 consisted of regulatory regions harboring motifs for Gata-s, Foxl2, Foxf1 and Foxo3, while C6 was highly enriched for Erg, Ets and Etv TFs (Figure 3E). C7 was specifically enriched in motifs for the immediate-early genes encoding members of the AP-1 TF family Batf, Fra1 and Fos (Figure 3E). AP-1 was shown to promote sarcomere disassembly and CM protrusion during zebrafish heart regeneration (50), consistent with C7 factors enriched at genes involved in actin filament organization. Protein network analysis of the TFs with enriched motifs within the different genomic regions associated with the A_Ia transition revealed NANOG, GATA4 and HOXA9 at the center of the regulatory network, which included TFs such as SOX, GATA, MEF2 and RUNX TFs (Figure 3F, Supplementary Table S3). Clusters C1, C5 and C8 were closely together and were specific for the U_Ia group, with GATA2, ISL1, ETV4 and BCL6 in the center of the protein network associated with U_Ia transition (Figure 3D, E, G, Supplementary Table S3). PITX1, GFI1B and GSC motifs were found in the largest C1 cluster, while EWSR1, FLI1 and ETVs were enriched together with ISL1, SCL and GFI1B in cluster 5 (Figure 3E). In contrast, C2 associated with cell division and was exclusively confined to the U_Pa group (Figure 3D, Supplementary Table S3). CDX2, FOXA1 and FOXO3 were predicted to be central for the U_Pa chromatin state switch at 1 dpci, with other major players MEIS1, MYB, FOXO-s, etc. (Figure 3D, E, H).

In stark contrast to 1 dpci, a similar analysis using 4 dpci as a reference point revealed higher chromatin state dynamics. Most of the chromatin state transitions were associated with a gain of active chromatin marks (Figure 4A). The Ia_A group was enriched in genes associated with actin filament organization, muscle cell differentiation, Wnt signaling, and heart left/right asymmetry (Figure 4B). This transition was predicted to be controlled by three TF ensembles sharing binding sites for Sox, Tead TFs, Mef2b and clock-related TFs (Clock, Bmal1), with specific enrichment of Ets1/Etv1 and Erg TFs for C7 and Fos, Fosl1, JunB, and Batf for C9 (Figure 4C–E, Supplementary Table S4). Regulatory network analysis suggested that CLOCK, NANOG, FOS and ERG act as central players (Figure 4E). The A_Ia group showed high chromatin state dynamics and was enriched for genes involved in the negative regulation of signaling and cell fate commitment. This dynamic group contained binding sites for three separate TF ensembles with Clock-related TFs (NPAS, BMAL1), FOS family members and SOX9 as central players (Figure 4C, D, F, Supplementary Table S4). Unmarked enhancer regions, which gained active histone marks (U_Ea), were associated with genes involved in heart morphogenesis, and cell junction organization, while unmarked promoter regions, that transitioned to an active promoter (U_Pa), were enriched for genes involved in chromatin organization, RNA-splicing, and cell maturation. The U_Pa group contained binding sites for two TF ensembles with C4 specifically enriched for Myb and Fox TFs, such as Foxf1, Foxk1, Foxp1, Foxl2 and Foxo3 (Figure 4D) and protein network analysis identified EOMES, FOXP1 and FOXO1 as the highest cooperativity proteins (Figure 4G).

Using 14 dpci as a reference we found that more than 60% of the regulatory regions switched from active to repressed chromatin state at 14 dpci (A_Ia) and were enriched in small GTPase signal transduction, actin filament organization and muscle cell differentiation (Figure 5A, B). Interestingly, these groups of genes were characterized with repressive marks at 1 dpci, followed by activation at 4 dpci and repression at 14 dcpi, underlying the dynamics chromatin changes during injury response (Figures 3–5). As central players associated with the A_Ia transition we identified CLOCK, NANOG, GATA2, FLI1, FOS and ETS1 TFs (Figure 5C–F, Supplementary Table S5). Many unmarked regulatory regions gained repressive characteristics (U_Ia) and were associated with genes involved in regionalization, Notch signaling pathway, and cell population proliferation (Figure 5A, B). These regions were highly enriched for RARA, ERG, ETS/ETV TFs, with SOX2, ETS1 and EWSR11 in the center of the regulatory network (Figure 5C, D, G, Supplementary Table S5). On the other hand, *cis-*regulatory elements which transitioned to an active chromatin state were enriched for genes involved in cardiac ventricle morphogenesis, wound healing, mRNA procession, chromatin organization, and ubiquitin-dependent protein catabolic processes. Unmarked regulatory regions that gained active marks at the promoter area showed enrichment for Myf5, Eomes and Fox TF motifs (Figure 5C, D, H).

Together, these analyses revealed highly dynamic chromatin states at specific subsets of genes and pinpointed putative upstream TF ensembles involved in chromatin state transitions during heart regeneration.

### Conservation of regulatory regions, upstream factors and cellular players in cardiac regeneration

**Figure 6.**
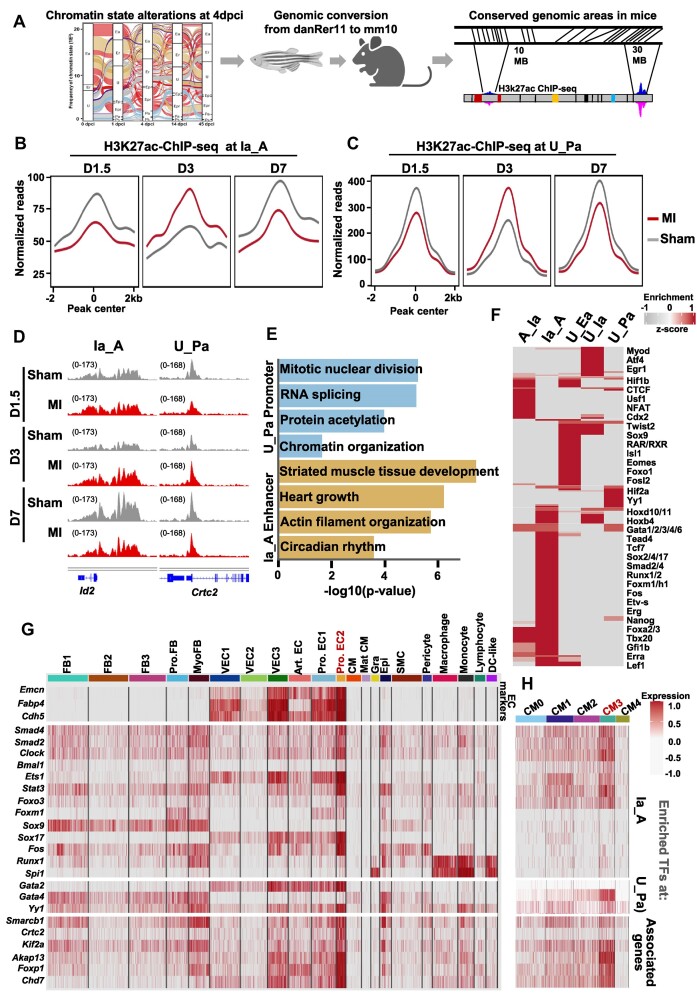
Conservation of chromatin state transitions between zebrafish and neonatal mice after heart injury. (**A**) Schematic representation of the experimental flow to identify converted regulatory regions in zebrafish (danRer11) and mouse (mm10) genomic areas. (**B, C**) Average H3K27ac ChIP-seq profiles of neonatal hearts at different time points (day D1.5, D3 and D7) after inducing myocardial infarction (MI) at 1 day post birth (P1) at conserved regions showing Ia_A (B) and U_Pa transitions at 4 dpci (C) (7,8) (GSE123868). (**D**) Genome tracks of H3K27ac ChIP-seq reads of neonatal hearts at different time points after MI at representative genes. (**E**) Enriched GO terms in genes associated with the U_Pa (cyan) and the Ia_A chromatin state transitions (beige). The P-value was determined by enrichGO from clusterprofiler after the ORA test; *P*-value *P < 0.05. (**F**) Heatmap displaying the enriched DNA binding proteins associated with the conserved chromatin state transition groups. (**G**) Heatmap displaying the expression of transcription factors associated with conserved Ia_A, U_Ea and U_Pa chromatin state transitions, and downstream regulatory genes at D3 after MI at P1 in single-cell RNA sequencing datasets from neonatal hearts (GSE153481). (**H**) Heatmap displaying the expression of transcriptional factors associated with conserved Ia_A, U_Ea and U_Pa chromatin state transitions, and downstream regulatory genes at D3 after MI in CM single-cell data (GSE130699).

We next studied, whether the dynamic enhancer and promoter areas in zebrafish might be conserved in mice. First, we used the LiftOver tool to convert the genomic coordinates of regions showing chromatin state transition at 4 dpci (Figure 4A) to the mouse mm10 (Figure 6A). Promoters that gained active chromatin marks showed the highest percentage of conservation (Supplementary Figure S5A and S5B). Next, we plotted H3K27ac ChIP-seq datasets from neonatal mice at day 1.5, day 3 and day 7 after myocardial infarction (MI) induced at postnatal day 1 (P1) and sham controls (8) (Figure 6B, C). Importantly, we observed a remarkable similarity to the chromatin state dynamics observed at these regions in zebrafish and mouse, i.e. an initial decrease of H3K27ac at day 1.5, followed by an increase at day 3 and a decrease at day 7 (Figure 6B, C). Genes associated within the conserved transition to active promoters (U_Pa) were associated with mitotic nuclear division, RNA-splicing, and chromatin organization, whereas inactive regions that gained active chromatin marks were enriched in striated muscle tissue development, heart growth, actin filament organization, and circadian rhythm (Figure 6D, E). Motif enrichment at the conserved regulatory regions predicted specific groups of TFs to induce the distinct chromatin state transitions (Figure 6F). Inactive regions that gained active chromatin marks were enriched Nanog, Sox TFs including Sox2, Erg, Fos, Etv-s, Runx1/2 Smad-s, Fox and Gata TFs, similar to the identified key regulatory factors of the Ia_A transition in zebrafish heart at 4 dpci. Unmarked promoters that become active (U_Pa) had significant enrichment of Hif2a, Yy1, Fli1 and Scl. Unmarked enhancers that become active showed enrichment of Hif1b, RAR/RXR, Isl1, Eomes and Foxo1. Interesting, genes associated with mesenchymal activation such as Twist2 and Sox9 were enriched at regions that acquired either active or repressive histone marks, similar to the analysis in zebrafish (Figure 6F).

To assess the possible involvement of these TFs in the different cardiac cell types during the regenerative process, we analyzed single-cell RNA-seq datasets of non-myocyte and CM cell populations at day 1 and 3 after MI injury (7,8) (Supplementary Figure S5C-E). Interestingly, we observed the highest expression of the selected top enriched conserved regulatory factors and associated genes in subpopulation of proliferative *Mki67* positive endothelial cells highly marked by endomucin (*Emcn*) and Fabp4, and expressing *Nfatc1*, *Nrp1* and *2*, *Eng*, *Dll4*, etc (Figure 6G). In CMs, conserved regeneration-associated TF assemblies, were highly enriched in subpopulation of proliferative CMs, marked by the expression of *Mki67*, *Gata4*, *Malat1*, *Rbm20* and *Camk2d* (CM3), which was increased by heart injury and expressed high levels of cyclin-dependent kinases (CDK) involved in cell cycling (Figure 6H, Supplementary Figure S5E, Supplementary Table S6).

Together, these results demonstrate a significant conservation of *cis-*regulatory elements, upstream factors and cellular players between zebrafish and neonatal mouse heart regeneration.

### TF activities driving cardiomyocyte dedifferentiation and proliferation

**Figure 7.**
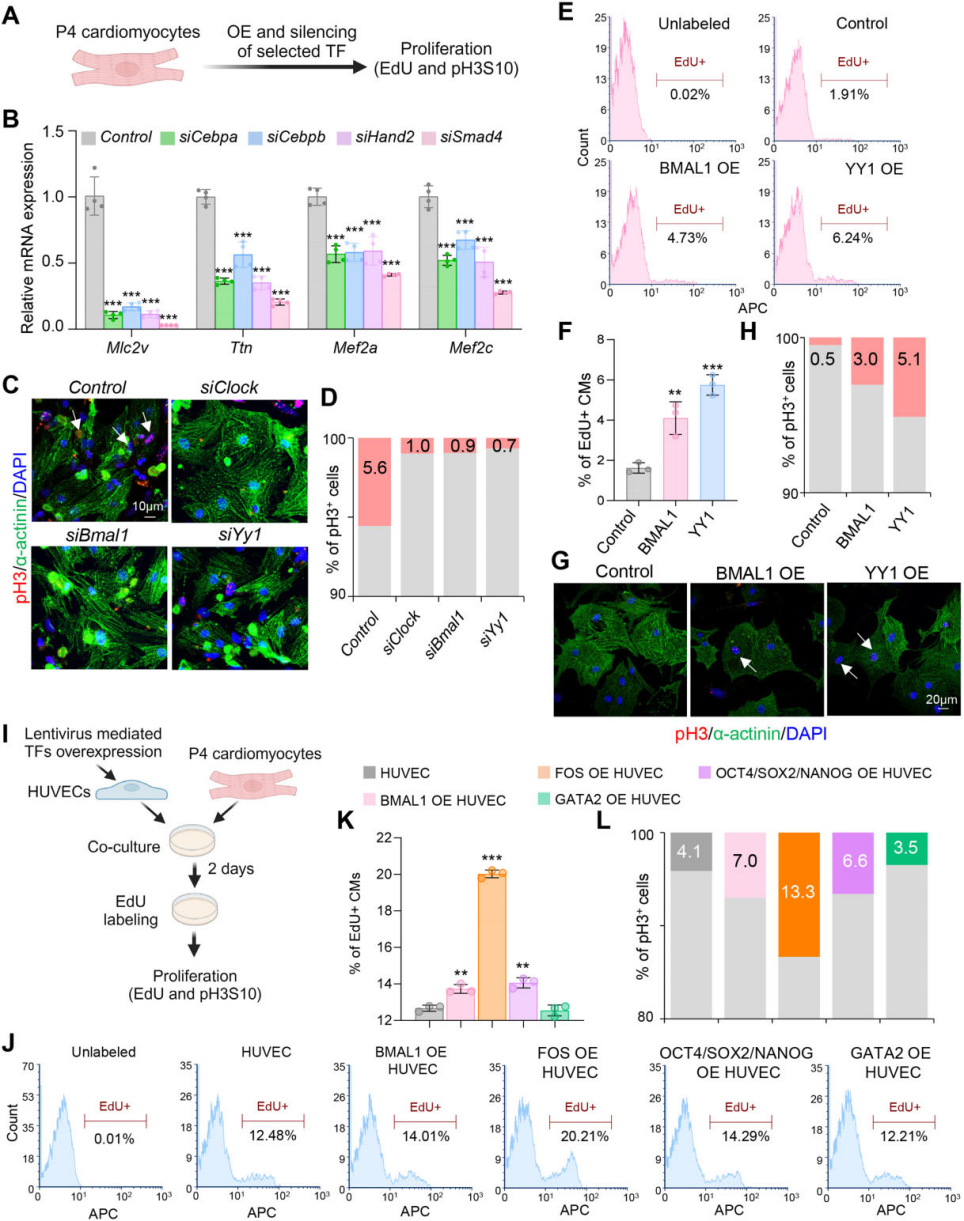
Functions of identified TFs in CM dedifferentiation and proliferation. (**A**) Schematic representation of the experimental setup for A–H. (**B**) Real-time PCR analysis of CM marker genes in P4 CMs after TF silencing. *n* = 4 biological replicates. (**C, D**) Representative immunostainings for the mitotic marker phospho-histone H3 (pH3S10) (red), α-actinin (green) and DAPI (blue) of P4 CMs, after siRNA mediated silencing of the indicated in the figure TFs (C) and quantification of pH3^+^ CMs (D). 200–300 cells were quantified in D. Arrows indicate pH3-positive (pH3^+^) cells. Scale bar: 10 μm. (E, F) Representative histograms showing FACS analysis of EdU^+^ cells after overexpression of human BMAL1 and YY1 in P4 CMs (**E**) and quantification of the percentage of EdU + CMs (**F**). *n* = 3 biological replicates. (G, H) Representative immunostainings of control, BMAL11 or YY1 overexpressing P4 CMs for the mitotic marker pH3S10 (red), α-actinin (green) and DAPI (blue) (**G**) and quantification of pH3^+^ CMs (**H**). 200–300 cells were quantified. Arrows indicate pH3^+^CMs cells. Scale bar: 10 μm. (**I**) Schematic representation of the co-culture of P4 CMs with HUVECs overexpressing control construct or human BMAL1, FOS, GATA2 alone, or OCT4, SOX2 and NANOG in combination, followed by subsequent analysis. (J, K) Representative histograms showing FACS analysis of EdU^+^ P4 CMs after 48 hours of co-culture with HUVECs overexpressing the indicated in the figure TFs (**J**) and quantification of the percentage of EdU^+^ CMs (**K**). *n* = 3 biological replicates. (**L**) Quantification of pH3 + P4 CMs after 48 h of co-culture with HUVECs overexpressing the indicated in the figure TFs. 200–300 cells were quantified. Data are presented as mean ± SD. Differences between groups were assessed using an unpaired two-tailed Student's *t*-test. *P< 0.05, **P< 0.01, ***P< 0.001.

As proof-of-principle, we next selected TFs identified in our analysis, based on their enrichment in the dynamic clusters, and tested their potential to stimulate CM dedifferentiation (*Cebpa*, *Cebpb*, *Hand2* and *Smad4*) or proliferation (*Clock*, *Bmal1* and *Yy1*). We first silenced or overexpressed selected TFs in postnatal day 4 (P4) mouse CMs, a stage associated with the beginning of CM cell cycle withdrawal (Figure 7A). Silencing of *Cebpa*, *Cebpb*, *Hand2*, and *Smad4* led to a significant decrease in CM markers gene expression (Figure 7B, Supplementary Figure S6A). On the other hand, silencing of *Clock*, *Bmal1* and *Yy1* led to a major decrease in mitotic (pH3S10-positive) CMs (Figure 7C, D), while overexpression of human BMAL1 and YY1 significantly increased the percentage of cycling (EdU-positive) and mitotic CMs (Figure 7E–H), supporting an important role of these TFs in CM proliferation during heart regeneration.

As we observed high expression of the top-enriched conserved regulatory factors and associated genes in a subpopulation of proliferative endothelial cells (Figure 6G) at day 3 after heart injury in mice and 4 dpci in zebrafish (Supplementary Figure S6B), we next tested whether their overexpression in endothelial cells might affect CM proliferation (Figure 7I). Consistent with a role of the endothelium in CM proliferation, co-culture of CMs with human umbilical vein endothelial cells (HUVECs) significantly increased the percentage of cycling and mitotic CMs (Supplementary Figure S6C–E). Furthermore, overexpression of human BMAL1, FOS, as well as a combination of OCT4/SOX2/NANOG in HUVECs significantly increased the percentage of proliferative CMs, while overexpression of GATA2 did not have an effect (Figure 7J–L). Particularly, the overexpression of FOS increased the percentage of mitotic CMs by more than three-fold (Figure 7L).

## Discussion

In this study, we have characterized the chromatin state transitions during cardiac regeneration and provided insights into how gene expression patterns are coordinated. We uncovered that the swift transcriptional response responsible for extracellular matrix reorganization and TOR signaling depends on the utilization of super-enhancers. The extent of H3K4me3, on the other hand, is strongly associated with the transcriptional activity of genes involved in ubiquitin-protein catabolic processes, proteolysis, cell cycle activity, and cell differentiation. Interestingly, changes in broad H3K4me3 regions preceded the deposition of H3K27ac, suggesting that changes in H3K4me3 breadth could be a reliable tool for the identification of TFs involved in the primary transcriptional response. These dynamic H3K4me3 changes occurring were overrepresented for YY1, CDX2, EST1, E2F4, HIF1A, GATA4 and SOX TF binding sites. Some of these factors have been shown to regulate CM dedifferentiation and/or proliferation, such as HIF1A, SOX and GATA TFs (51–54). YY1 plays an important role in early heart development (55) and a protective role in cardiac remodeling after myocardial infarction (56). It has also been implicated in stimulating skeletal muscle regeneration through metabolic rewiring (57). Importantly, YY1 overexpression elevated the percentage of mitotic CMs 10-fold, supporting the notion that Yy1 may play an important role in cardiac regeneration. Recently, multipotent fetal placenta-derived Cdx2+ cells were shown to spontaneously differentiate into beating CMs (CMs) and vascular cells and improve cardiac repair after MI (58), suggesting that Cdx2 activation might be important for the cardiac regenerative potential. Thus, overexpression of these TF ensembles together with trithorax-group proteins, responsible for catalyzing H3K4me3 might stimulate cardiac regeneration.

Following H3K27ac dynamics, we observed that the rapid transcriptional activation of genes involved in blood vessel development and proteostasis upon myocardial injury involves the engagement of super-enhancers by SPI1, MYCN, TEAD and RUNX TFs, which play an important function in endothelial cells but also in endothelial-to-hematopoietic transition (EHT) (59–61). Fast revascularization is essential to support zebrafish heart regeneration (62), but whether injury can induce EHT and whether EHT plays a role in regulating the regenerative potential of the heart is largely unexplored and could be an exciting area of study in the future. CDX2, NANOG, FOX, GATA and SOX TFs were enriched at genes that showed increased H3K27ac already at day 1 and a peak at day 4 after heart injury. These genes were involved in ECM organization and TOR signaling and pointed to CDX2 and NANOG as central players. Indeed, recent studies revealed that the ECM promotes heart regeneration and that changes in ECM stiffness may limit this capacity to the first days after birth (63,64). Amino-acid-driven mTOR signaling was also shown to be required for CM proliferation (65). Thus, the above-mentioned TF ensemble might stimulate dedifferentiation and CM proliferation by inducing rapid ECM reorganization and mTOR-mediated metabolic rewiring, via promoting histone acetylation at these genes. Identifying histone acetyltransferases that play a role in this context would be of great importance. We also observed a cluster of genes which showed opposite dynamics, i.e. decreased H3K27ac already at 1 dcpi and a major dip at 4 dpci. This cluster was enriched in SMAD4, HAND2 and CCAAT Enhancer Binding Protein Alpha (CEBPA) binding sites, suggesting that CM dedifferentiation might be stimulated by lowering the activity of these TF assemblies. Proof-of-principle experiments demonstrated that the silencing of *Smad4*, *Hand2*, *Cebpa*, and its paralog *Cebpb* does indeed result in a significant decrease in CM gene expression.

By analyzing chromatin state transition during the different time points of cardiac regeneration we identified an extensive accumulation of repressive chromatin marks one day after myocardial injury, followed by a large-scale acquisition of active chromatin characteristics at day 4 and a switch to a repressive state at day 14. These state transitions were associated with specific subsets of genes and controlled by either gain or loss of function of distinct TF ensembles. Within these ensembles were TFs that have been shown to regulate cardiac regeneration, such as GATA4/6, JUN/FOS (AP-1), TEAD, SOX TFs, including SOX2, NANOG, OCT4, TBX5, etc. Interestingly, NANOG, CLOCK, FOS and ERG were found as central players in inactive to active chromatin state transitions. *Fos* expression was highest in endothelial cells expressing the endocardial marker *Emcn* and the coronary endothelial marker *Fabp4*. Indeed, the overexpression of FOS in endothelial cells increased the percentage of mitotic cardiomyocytes in a co-culture system by >3-fold, supporting an important role of the AP-1 complex (Fos/Jun) not only in CMs (50) but also in endothelial cells for CM proliferation and cardiac regeneration. Recently, Nanog was shown to be activated upon hypoxia and to promote neuronal regeneration in response to ischemic stress (66), suggesting that such a mechanism might me exploited in myocardial injury. In fact, there is a functional link between cellular reprogramming and regenerative ability, as heart-specific expression of Oct4, Sox2, Klf4 and c-Myc (OSKM) induces adult CM dedifferentiation and cardiac regeneration (14). Thus, activation of Nanog, Sox2, Oct4 and Myb (activates c-myc expression) upon cardiac injury might be a crucial mechanism conferring the robust regenerative potential of the zebrafish heart. Interestingly, we observed a major increase of Oct4 and Nanog in the endocardium within the injured area. Further, overexpression of OCT4, NANOG and SOX2 in endothelial cells significantly increased CM proliferation in a co-culture system, supporting a crucial function of these factors also in endocardial/ endothelial cells for heart regeneration.

Two central factors predicted in our study are CLOCK and BMAL1, TFs involved in the circadian clock (CK). CK influences heart contractility by affecting the function of the different cardiac cell types. In CMs, CK promotes oxidative metabolism at the sleep-wake transition, which facilities the renewal of the myocardium before awakening (67). Interestingly, there is a strong correlation between SNPs in *Clock* and the incident of myocardial infarction, supporting its important role in heart homeostasis (68). Interestingly, Clock was specifically associated with the chromatin state transitions in zebrafish. In contrast to neonatal mice in which the regenerative ability might be due to the proliferative state of CMs in the neonatal heart (5,7,8), cardiac regeneration in adult zebrafish involves CM cell cycle re-entry. Importantly, silencing of *Clock* and *Bmal1* reduced CM proliferation, while overexpression of BMAL1 in CMs and endothelial cells significantly increased the percentage of cycling and mitotic CMs. Given the major involvement of the CK-related proteins in the dynamic chromatin state transitions in adult zebrafish heart regeneration, understanding the role of the CK in unlocking the regenerative potential of the human heart will be of key importance. A large number of members of the forkhead box (Fox) TF family were also associated with the transition from inactive to active chromatin state. FOX TFs play a key role in the control of cell cycle, metabolism, differentiation, and aging and are instrumental for cardiovascular development and homeostasis (69). Thus, exploring their combinatorial activities and cooperativity in heart regeneration using functional studies will be important. In addition to factors that regulate CM behavior, we found TFs critical for neurogenesis and immune cell and fibroblast activation, such as Nifl3, Irf4, Tbr1, Neurog1/2, Otx2, Sox9, Runx2, Tgif1/2, etc. Defining their function in heart regeneration and their role in the dynamic contribution of the different cell types in the regenerative process will be an important direction to follow.

Finally, we detected significant evolutionary conservation between the regulatory regions that drive zebrafish and neonatal mouse regeneration which were associated with conserved TF assemblies, suggesting that the reactivation of transcriptional and epigenetic networks converging on these conserved elements might induce the regeneration capacity of the mammalian heart. Interestingly, the highest conservation was detected in regulatory sequences that gained active histone marks. This could be due to the distinct mechanisms of inheritance of active and repressive histone marks (70). While repressive histone marks have readers that can mediate their self-reestablishment after genome duplication, active histone marks require the binding of TF and thereby conserved sequences. Some of the identified conserved TF assemblies implicated in heart regeneration were highly enriched in subset of proliferative endothelial cells after MI. Our data revealed that overexpression of BMAL1, FOS or a combination of OCT4, NANOG and SOX2 in endothelial cells is sufficient to significantly increase CM proliferation. In this context, endothelial cells and in particular endocardial endothelial cells have been shown to serve as an important signaling center for heart regeneration. For example, activation of Bmp signaling, reflected by phosphorylation of Smads is also observed in endocardial cells and CMs in injured zebrafish heart and Bmp inhibition impairs regeneration, whereas overexpression of the Bmp ligand *bmp2b* promotes regeneration (71). Smad motifs were highly enriched at conserved regulatory elements, supporting the validity of our approach. Ligands and receptors involved in the Insulin-like Growth Factor (IGF) signaling are also altered in endocardial cells and CMs upon cardiac injury. Blocking IGF signaling impairs heart regeneration, while treatment with an IGF signaling agonist is beneficial (72,73). We identified TFs involved in IGF signaling, such as Stat3 and Foxo TFs to be enriched at conserved regulatory elements supporting heart regeneration, further endorsing the notion that activation of TF assemblies promoting the endocardial/endothelial-CM crosstalk in the heart might be essential for efficient cardiac regeneration.

Altogether, this study is the first to provide a genome-wide view of histone code dynamics during zebrafish heart regeneration and to provide insights into the regulatory networks that have the potential to break through the epigenetic barrier hindering heart regeneration.

## Supplementary Material

gkae085_Supplemental_Files

## Data Availability

All ChIP-seq data in this study have been deposited in the GEO repository under the accession number GSE211677. Codes have been deposited in Zenodo at https://zenodo.org/records/10566908, and in GitHub (https://github.com/jcorder316/01TFinZONE), and are also available upon request to the corresponding authors.
