# Supplementary material for: Leveraging chromatin state transitions for the identification of regulatory networks orchestrating heart regeneration
Source: Nucleic Acids Res. 2024 Feb 14;52(8):4215–33. doi: 10.1093/nar/gkae085 (PMC11077086; doi:10.1093/nar/gkae085)
Supplement: gkae085_Supplemental_Files [file gkae085_supplemental_files.zip › Suplementary table captions.docx]

**Supplementary table captions**

**Supplementary Table S1.** H3K27ac dynamics in zebrafish heart regeneration. (A) List of H3K27ac Chip-seq peaks with normalized values per time point and annotation of peaks to genes. (B) Genes in dynamic clusters and cluster’s identifier (ID).

**Supplementary Table S2.** H3K4me3 dynamics in zebrafish heart regeneration. (A) List of H3K4me3 Chip-seq peaks with normalized values per time point and annotation of peaks to genes. (B) Genes in dynamic clusters and cluster’s identifier (ID).

**Supplementary Table S3.** Top transcription factors enriched in clusters containing genomic regions with similar chromatin state one day after heart injury.

**Supplementary Table S4.** Top transcription factors enriched in clusters containing genomic regions with similar chromatin state four days after heart injury.

**Supplementary Table S5.** Top transcription factors enriched in clusters containing genomic regions with similar chromatin state fourteen days after heart injury.

**Supplementary Table S6.** Cell identities and top differentially expressed genes in single-cell RNA-seq data reanalyzed from GSE153480 (A) and GSE130699 (B).
