# Supplementary material for: Leveraging chromatin state transitions for the identification of regulatory networks orchestrating heart regeneration
Source: Nucleic Acids Res. 2024 Feb 14;52(8):4215–33. doi: 10.1093/nar/gkae085 (PMC11077086; doi:10.1093/nar/gkae085)
Supplement: gkae085_Supplemental_Files [file gkae085_supplemental_files.zip › Supplementary Data.pdf]

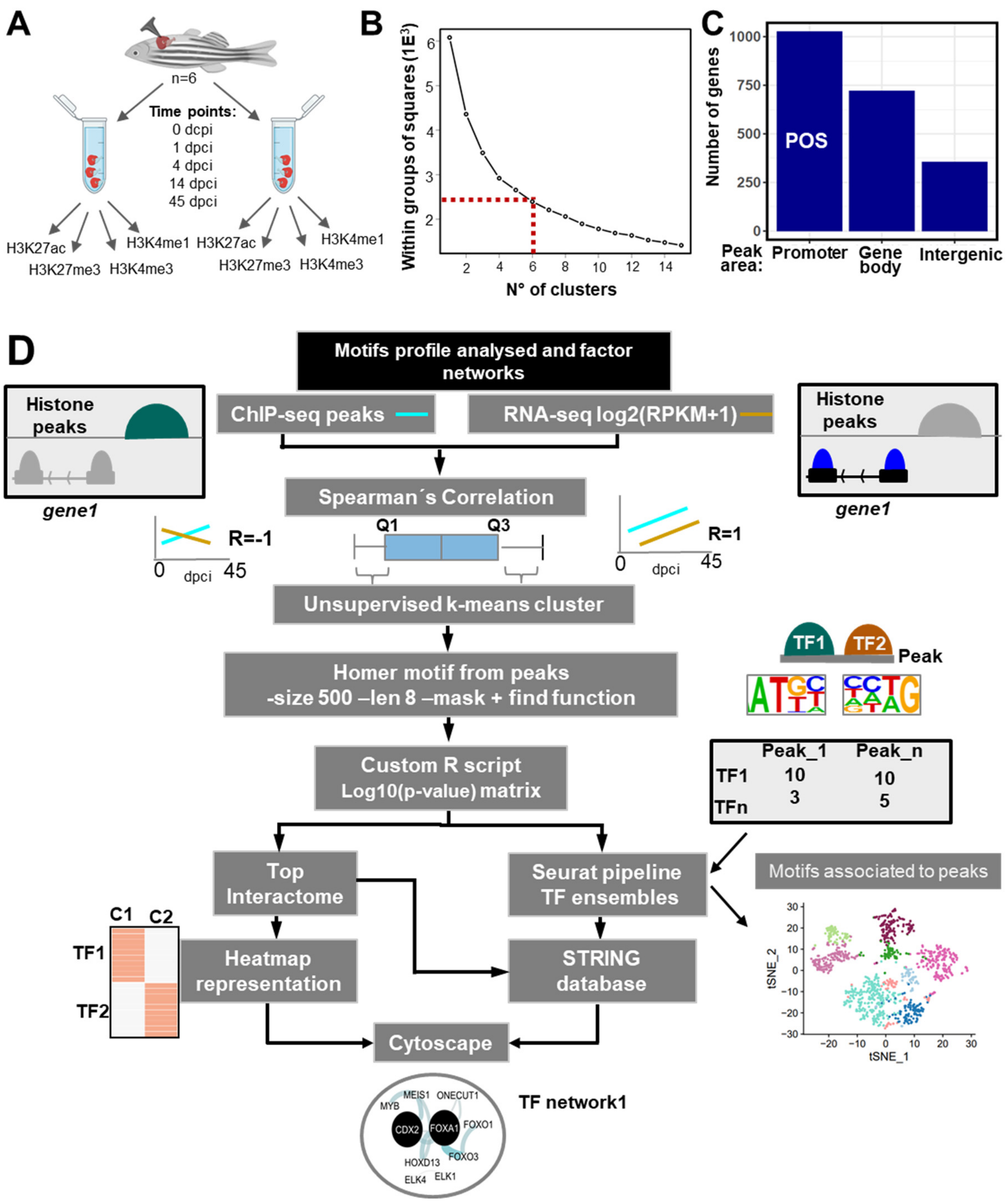

Supplementary Figure S1. **H3K27ac dynamics in zebrafish heart regeneration. (A)** Schematic representation of the experimental setup. **(B)** Elbow plot of total within-cluster sum of squares to identify the optimal number of clusters representing H3K27ac dynamics during regeneration. **(C)** Bar plot showing the number of H3K27ac peaks positively correlated to the neighboring gene after Spearman's rank correlation coefficient test (rho>0.3 & p-value <0.25). **(D)** Workflow used to define enriched TFs and TF networks in regulatory regions.

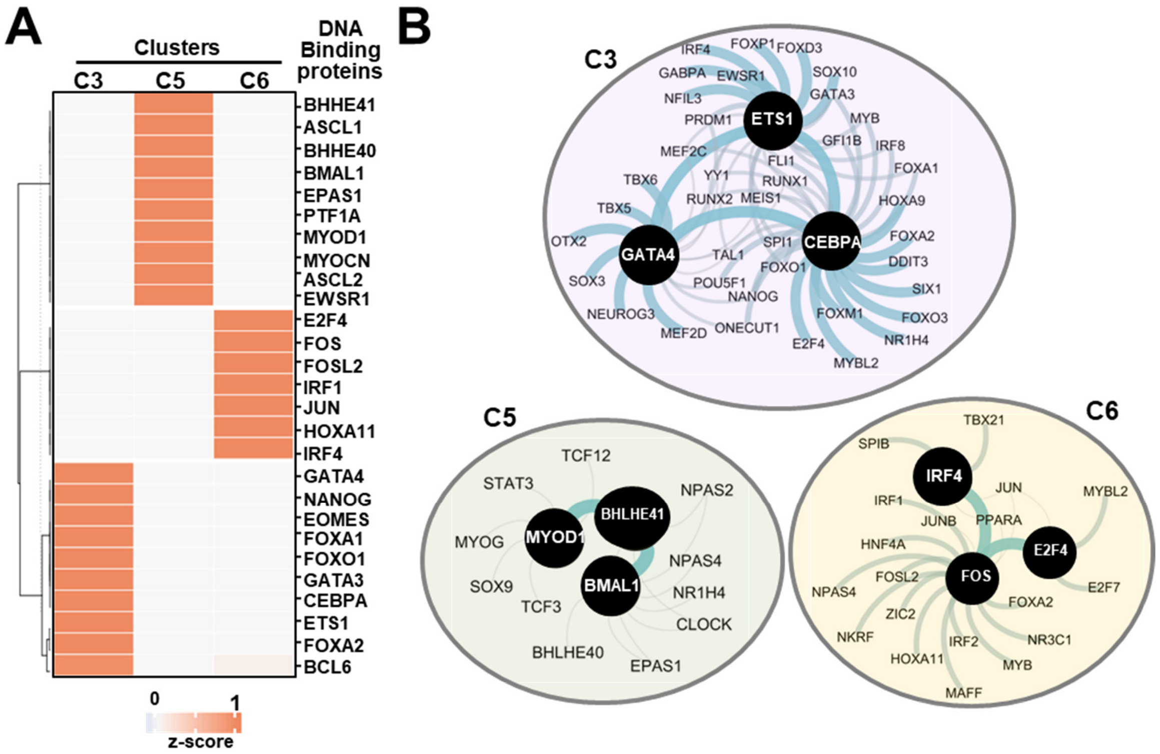

Supplementary Figure S2. Transcription factor motifs associated with H3K27ac dynamics during zebrafish heart regeneration. (A) Heatmap showing the TF motifs associated with the dynamic H3K27ac clusters 3, 5 and 6 (C3, C5; C6). **(B)** Interaction network of TFs identified by motif analysis of genomic regions within C3 (top panel), C5 (bottom, left panel) and C6 (bottom, right panel).

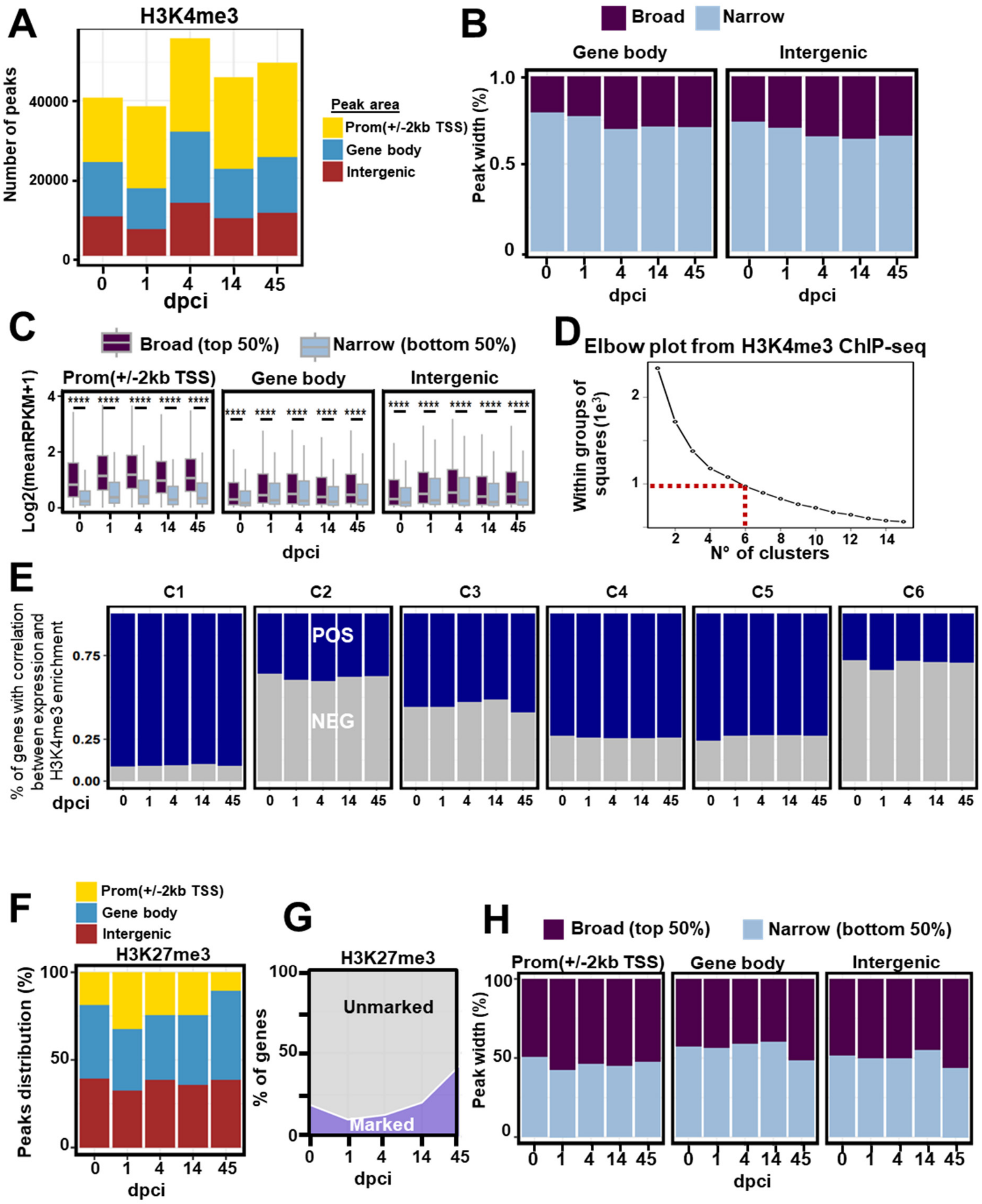

Supplementary Figure S3. Correlation of H3K4me3 and H3K27me3 broadness with gene expression. (A) Total amount of H3K4me3 peak per timepoint after peak calling using the MACS2 broad function. **(B)** Stacked bar plot showing the breadth of H3K4me3 peaks at different time points after heart injury. Broad peaks (>0.5kb; top 50%) (dark violet), and narrow peaks (<0.5kb; bottom 50%) (light blue). P-values **P < 0.001 calculated after Fisher`s exact test. **(C)** Boxplot showing the expression of genes associated with H3K4me3 peaks at

promoters, gene body or intergenic regions, separated by the broadness of the H3K4me3 peak. Values are the log2 from the mean of reads per kilobase million (RPKM)+1. ****P < 0.01 calculated after the two-sided Wilcox test. **(D)** Elbow plot of total within-cluster sum of squares to identify the optimal number of clusters representing H3K4me3 dynamics during regeneration. **(E)** Percentage of genes within dynamic H3K4me3 clusters positively and negatively correlating with gene expression. **(F)** Stacked bar plot showing the distribution of the H3K27me3 peaks at the promoter (TSS+/- 2kb) (yellow), gene body (TSS>2kb) (blue), and intergenic regions (maroon). Values are the percentage of each region at the different time points after cardiac injury. **(G)** Density plot representing the percentage of genes associated with H3K27me3 peaks (purple) or lacking the H3K27me3 mark (grey). **(H)** Distribution of H3K27me3 based on the broadness of the H3K27me3 peak. Broad peaks (>0.8kb; top 50%) (dark violet); narrow peaks (<0.8kb; bottom 50%) (light violet).

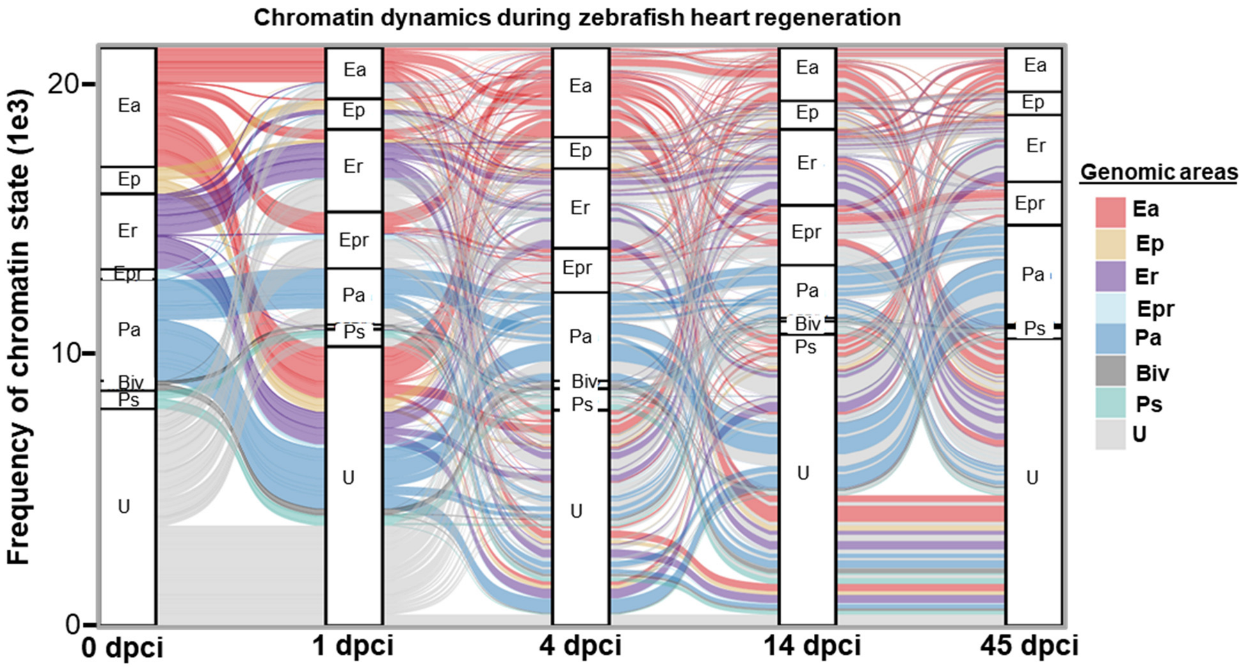

Supplementary Figure S4. Chromatin state dynamics during zebrafish heart regeneration. Alluvial plot representing the dynamics of chromatin states. Values are the frequency of the assigned chromatin states.

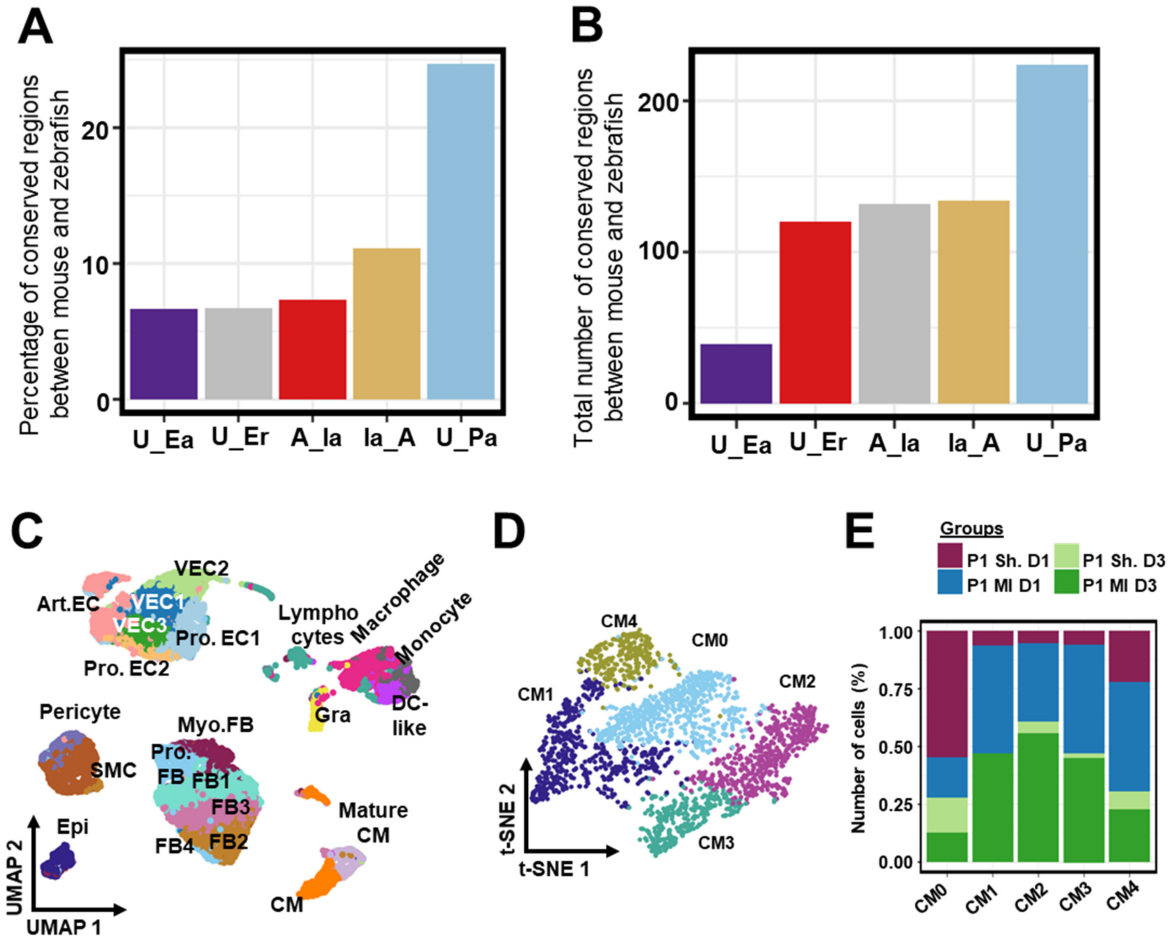

Supplementary Figure S5. Conserved in zebrafish and mouse genomic regions showing chromatin state transitions at 4 dpci and upstream regulatory TFs. (A) Bar plot representing the percentage of conserved genomic regions in zebrafish and mouse showing chromatin state transitions at 4 dpci. **(B)** Bar plot representing the total number of conserved in mouse genomic regions, characterized by distinct chromatin state transitions. **(C)** U-MAP plot displaying the different cell types found in single-cell RNA-seq data reanalyzed from GSE153480. Injury in the study was performed at P1 and single cell RNA-seq was performed one day (P1 Sham D1 and P1 MI D1) or 3 days (P1 Sham D3 and P1 MI D3) after MI (7,8). **(D)** t-SNE plot displaying the different cardiomyocyte clusters identified in cells expressing at least 1 count of any of the markers (*Tnnt2*, *Myh6*, and *Ttn*). Single-cell data are retrieved and reanalyzed from (GSE130699), containing datasets from day 1 (P1-D1) and day 3 (P1-D3) cardiomyocytes after MI in neonatal P1 mouse hearts. **(E)** Percentage of the different cardiomyocyte subpopulations at day 1 and day 3 after sham operation or MI.

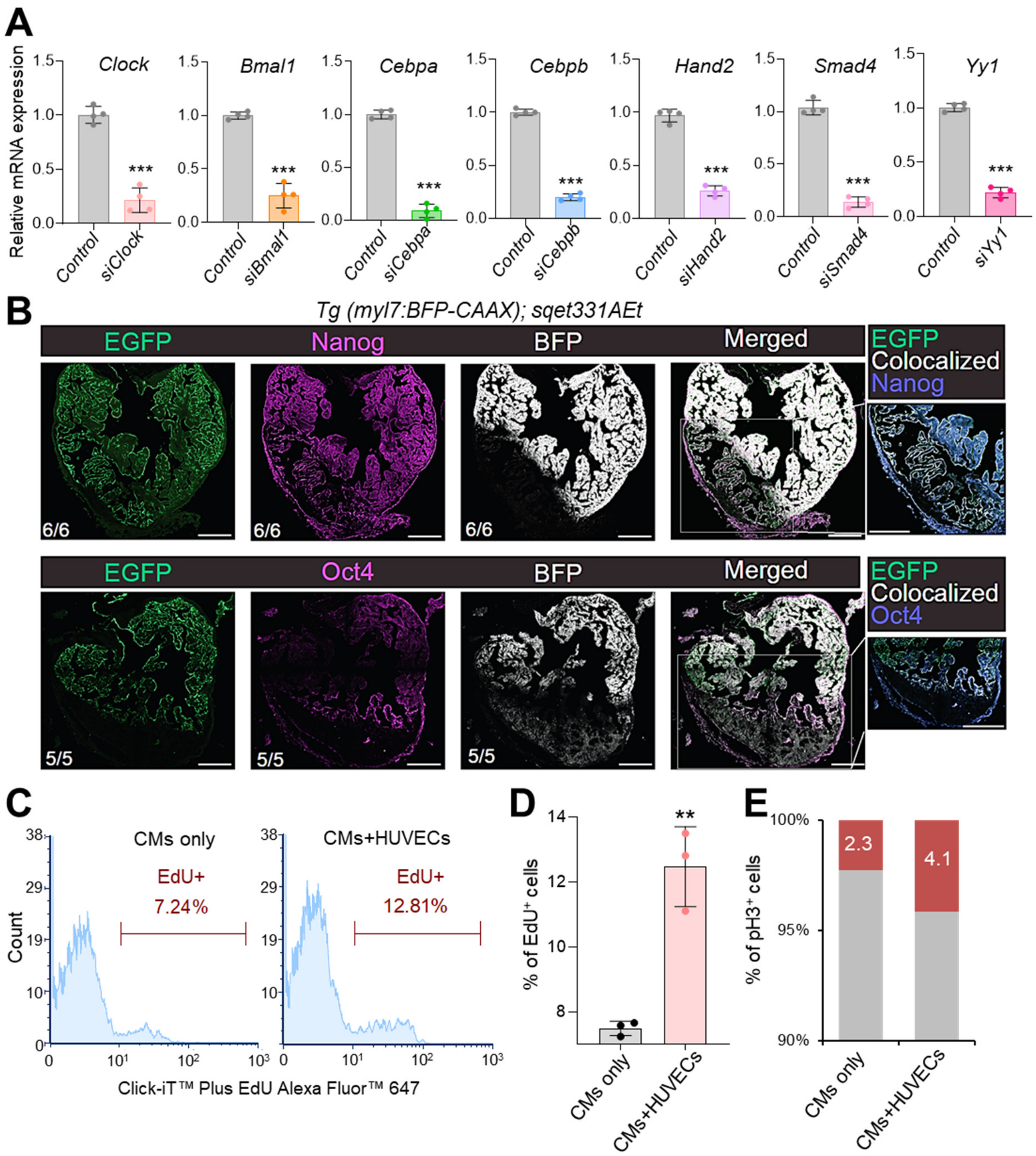

Supplementary Figure S6. Endothelial control of CM proliferation. (A) Relative TF mRNA expression in P4 CMs after TF silencing with siRNA (n=4 biological replicates). **(B)** Maximum intensity projection (Nanog) and single-plane (Oct4) confocal images of Tg(myl7:BFP-CAAX); sqet331AEt ventricles at 4 dpci for Nanog (n=6, top panels) and Oct4 (n=5, bottom panels) staining. White boxes indicate the areas shown in the zoomed image. Colocalized pixels are represented in white. Scale bar = 0,5 mm. **(C, D)** Representative histograms showing FACS analysis of EdU$^+$ P4 CMs cultured alone or together with HUVECs **(C)** and quantification of the percentage of EdU+ CMs **(D)**. n=3 biological replicates. **(E)** Quantification of pH3+ P4 CMs cultured alone or together with HUVECs. n=200-300 cells were quantified.
